# Contrasting Patterns in the Evolution of Vertebrate MLX Interacting Protein (*MLXIP*) and MLX Interacting Protein-Like (*MLXIPL*) Genes

**DOI:** 10.1371/journal.pone.0149682

**Published:** 2016-02-24

**Authors:** Parmveer Singh, David M. Irwin

**Affiliations:** 1 Department of Laboratory Medicine and Pathobiology, University of Toronto, Toronto, Ontario, Canada; 2 Banting and Best Diabetes Centre, University of Toronto, Toronto, Ontario, Canada; Laboratoire Oceanologique de Banyuls sur Mer, FRANCE

## Abstract

ChREBP and MondoA are glucose-sensitive transcription factors that regulate aspects of energy metabolism. Here we performed a phylogenomic analysis of *Mlxip* (encoding MondoA) and *Mlxipl* (encoding ChREBP) genes across vertebrates. Analysis of extant *Mlxip* and *Mlxipl* genes suggests that the most recent common ancestor of these genes was composed of 17 coding exons. Single copy genes encoding both ChREBP and MondoA, along with their interacting partner Mlx, were found in diverse vertebrate genomes, including fish that have experienced a genome duplication. This observation suggests that a single *Mlx* gene has been retained to maintain coordinate regulation of ChREBP and MondoA. The ChREBP-β isoform, the more potent and constitutively active isoform, appeared with the evolution of tetrapods and is absent from the *Mlxipl* genes of fish. Evaluation of the conservation of ChREBP and MondoA sequences demonstrate that MondoA is better conserved and potentially mediates more ancient function in glucose metabolism.

## Introduction

Carbohydrate metabolism is essential for life, with defects leading to diseases such as diabetes [1,2]. The metabolism of carbohydrate is regulated at multiple levels, including changes at the physiological, e.g., via hormones such as insulin and glucagon [3], enzymatic, e.g., regulation of enzyme cellular localization and activity [4–6], and genomic, e.g., gene expression [7,8] levels. Glucose is a primary carbohydrate in metabolism, as it can be used to generate energy or stored for future use [9]. Upon import of glucose into a cell, it is immediately phosphorylated to glucose-6-phosphate by glucokinase, or another hexokinase, to become a substrate for further metabolism [4,5,10]. The fate of glucose, to be metabolized for energy or stored as glycogen or lipid, depends upon the tissue and metabolic state of the host [5,8, 9]. The levels of the enzymes that metabolize glucose are regulated at both the transcriptional and post-transcriptional level [7,8]. Many transcription factors have been identified that regulate enzymes involved in energy metabolism [2,7,11].

Carbohydrates themselves regulate the expression of genes involved in metabolism [2,4,11]. Genes regulated by glucose possess carbohydrate response elements (ChoREs), which contain conserved consensus sequence composed of two E box elements separated by 5 nucleotides [12]. The basic helix-loop-helix leucine-zipper (bHLH-Zip) protein ChREBP (carbohydrate response element binding protein, also known as MondoB), an 852 amino acid long protein, was found to bind to this element and confer glucose-sensitivity to a number of promoters containing a ChoRE [13,14,15]. The gene encoding ChREBP has had multiple names, initially named *WBSCR14* as it was found in a deletion associated with Williams-Beuren syndrome [16,17], with its current gene name being *MLXIPL*. Transcriptional activation of gene expression by ChREBP requires the formation of a heterodimer with a second basic helix-loop-helix protein, the 244-amino-acid-long protein MLX (Max dimerization protein, also known as Max-like protein X and TCFL4, transcription factor-like 4, encoded by *MLX*) [18,19]. In addition to a bHLH-Zip domain, both ChREBP and MLX share a DCD (dimerization and cytoplasmic localization domain), a domain thought to have roles in protein dimerization and DNA binding [15,20]. ChREBP possesses a number of domains in its N-terminal sequences that are not present in MLX [15,20], including the LID (low glucose inhibitory domain) and GRACE (glucose responsive activation conserved element) domains, and a proline rich region in the middle of the protein. The GRACE domain confers transactivation ability to ChREBP, an activity that is regulated by the LID domain [21]. Under low glucose conditions, transactivation by the GRACE domain is repressed, with this inhibition released when glucose levels become elevated. Recently a second isoform of ChREBP, ChREBP-β, has been identified that lacks the LID domain (full length isoform being ChREBP-α) and thus constitutively has transactivation ability and is more potent [22,23].

While a single gene encoding a ChREBP-like (or Mondo) protein has been described in several non-vertebrate species, two paralogous genes have been found in several vertebrates, *Mlxipl*, which encodes ChREBP, and *Mlxip*, which encodes MondoA [15,24]. Among basic helix-loop-helix leucine-zipper protein genes, *MLXIP* and *MLXIPL* are each other’s closest relatives, and originated via the genome duplication event that occurred very early in vertebrate evolution, with *MLX* being the next most closely related gene, with a divergence that predates the separation of many major animal groups (i.e., before the divergence of insects and vertebrates) [24]. MondoA has a similar domain structure to ChREBP, and also interacts with Mlx to form a glucose-responsive transcription factor [15,20]. Despite the similarities between MondoA and ChREBP there are differences. Both have widespread expression, however ChREBP predominates in the liver where it regulates lipogenesis, while MondoA is most abundant in muscle and regulates the glycolytic pathway [15,19]. The two transcription factors regulate different sets of genes, which is not simply due to differences in their expression patterns, as each interacts with specific promoters. Evidence supporting differences in promoter regulation of these two transcription factors is derived from an experiment where ChREBP, but not MondoA, was found to rescue the glucose response in hepatocytes transfected with a dominant negative Mlx [25]. Comparisons of the amino acid sequences of vertebrate MondoA and ChREBP proteins, and non-vertebrate Mondo proteins have led to refinements of the boundaries of the conserved regions in the N-terminal extension of these proteins, with Mondo Conserved Regions (MCR1-6) identified within the LID and GRACE domains shared by ChREBP, MondoA and the non-vertebrate Mondo homologs [26–28]. Within and overlapping these domains, a number of functional elements have been identified, such as sequences for nuclear import and export signals and mitochondrial localization, some of which might be paralog specific [20]. The domains shared by ChREBP and MondoA likely explain many of their overlapping functions, however, differences in the functions of these proteins should lead to changes in the evolutionary constraints acting upon these sequences. Here we examined *MLX*, *MLXIP*, and *MLXIPL* genes from diverse vertebrate species to better understand the evolution of their sequences and identify sequences that might account for the difference in the functions of these genes.

## Materials and Methods

### Database searches

Human and mouse Max dimerization protein (Max-like protein X; gene symbol: *MLX*), Mlx interacting protein (MondoA; gene symbol: *MLXIP*), Mlx interacting protein-like (ChREBP, MondoB, WSCBR14; gene symbol: *MLXIPL*), and *Drosophila melanogaster* Mondo (Mlx interactor; gene symbol: *Mio*) coding, genomic, and protein sequences were downloaded from the Ensembl genome database (www.ensembl.org). Additional *Mlx*, *Mlxip*, and *Mlxipl* coding sequences and genes were identified from genome sequences maintained in the Ensembl database via similarity searches with the tblastn algorithm [29] using protein sequences encoded by the human genes as queries. Searches were conducted with the genomes that were available in the Ensembl database release 80 in May 2015. Additional blast searches were conducted with diverse Mlx, MondoA, ChREBP protein sequences identified by these searches. All sequences that had E-scores below 0.01 were examined. Sequences identified in the blast searches were used in reciprocal blastx searches of the human and mouse proteomes to ensure that their best matches were Mlx, MondoA, or ChREBP protein sequences. As some genomes did not yield all of the expected intact genes, the NCBI sequence database (www.ncbi.nlm.nih.gov) was also searched for a few sequences (see S1 Table).

Coding exons in the *Mlx*, *Mlxip*, and *Mlxipl* genes were identified from genomic alignments generated with MultiPipMaker [30,31]. Human gene sequences were used as master sequences with the locations of coding sequences and exons obtained from the Ensembl annotations. Repetitive elements in the human (and other) genes were identified using RepeatMasker [32]. Additional MultiPipMaker alignments were generated for genomic sequences if exons could not be identified when the human gene sequences were used as the master sequence. For these genomic alignments other species were used as the master sequence, where these genes contained all the coding exons and if possible were more closely related to the species with the missing exons. If these searches also failed to find the missing exons, then the gene annotations from Ensembl were examined to determine whether any appropriate exon had been predicted by the annotations. *Mlx* genes that contained all 8 coding exons and *Mlxip* and *Mlxipl* genes that had 17 coding exons were used to predict coding sequences for subsequent analyses.

### Protein coding sequence alignments and evolutionary analyses

Initial alignment of the human and mouse Mlx, MondoA, and ChREBP protein sequences and the *Drosophila melanogaster* Mondo protein sequence was generated using Clustal Omega (http://www.ebi.ac.uk/Tools/msa/clustalo/) [33]. Use of other aligners yielded similar alignments. Intron positions were mapped onto the multiple protein sequence alignment based on the annotations of the genes from the Ensembl database. Subsequent DNA alignments of *Mlx*, *Mlxip*, and *Mlxipl* coding sequences were generated at the codon level using MAFFT [34] as implemented on the Guidance web server (http://guidance.tau.ac.il) [35], using default parameters. Translating the DNA coding sequence alignments generated protein sequence alignments.

Phylogenetic trees of *Mlx*, *Mlxip*, and *Mlxipl* sequences were generated using Bayesian methods with MrBayes 3.2.2 [36], maximum likelihood with PhyML 3.0 [37], and neighbor-joining distance approaches with MEGA6.0 [38]. Bayesian trees were generated from the coding sequences using parameters selected by hierarchical likelihood ratio tests with ModelTest version 3.8 [39], as implemented on the FindModel server (www.hiv.lanl.gov/content/sequence/findmodel/findmodel.html). MrBayes was run for 2,000,000 generations with four simultaneous Metropolis-coupled Monte Carlo Markov chains sampled every 100 generations. The average standard deviation of the split frequencies dropped to less than 0.02 for all analyses. The first 25% of the trees were discarded as burn-in with the remaining samples used to generate the consensus trees. Trace files generated by MrBayes were examined by Tracer (tree.bio.ed.ac.uk/software/tracer/) to verify that they had converged. Bootstrapped maximum likelihood trees, 100 replications, were generated with PhyML [37] on the PhyML webserver (www.atgc-montpellier.fr/phyml/) using parameters for the substitution model suggested by ModelTest. Maximum likelihood searches was initiated from trees generated by BIONJ and the best tree was identified after heuristic searches using the nearest neighbor interchange (NNI) algorithm. MEGA6.0 [38] was used to construct bootstrapped (1000 replications) neighbor-joining distance trees, using either Maximum Composite Likelihood distances for the DNA sequences or JTT distances for the protein sequences. Choice of alignment method (MAFFT [34] or Clustal Omega [33]), or the use of full-length or trimmed (based on Guidance scores [35]) alignments had little influence on the key findings of these analyses. Methods that relied on shorter sequences (i.e., trimmed alignments or protein sequences) or simpler models of sequence evolution (i.e., neighbor-joining) tended to yield weaker support for the earlier diverging lineages, but none of our analyses were in significant conflict with the key inferences of our inferred phylogenies.

Conservation of proteins sequences was assessed using two measures. Conservation within a protein alignment was measured using Jenson-Shannon (JS) divergence scores (http://compbio.cs.princeton.edu/conservation/) [40], using a window size of 3 and the BLOSUM62 matrix as background. In addition, pairwise protein sequence differences (p-distance) distances were measured for the entire protein, or protein domains, using MEGA6.0 [38]. Consensus sequences of domains within the protein sequences were generated using Weblogo (http://weblogo.berkeley.edu/logo.cgi) [41].

## Results and Discussion

### Structure of the gene for the ancestor of *Mlxip* and *Mlxipl*

**Fig 1:**
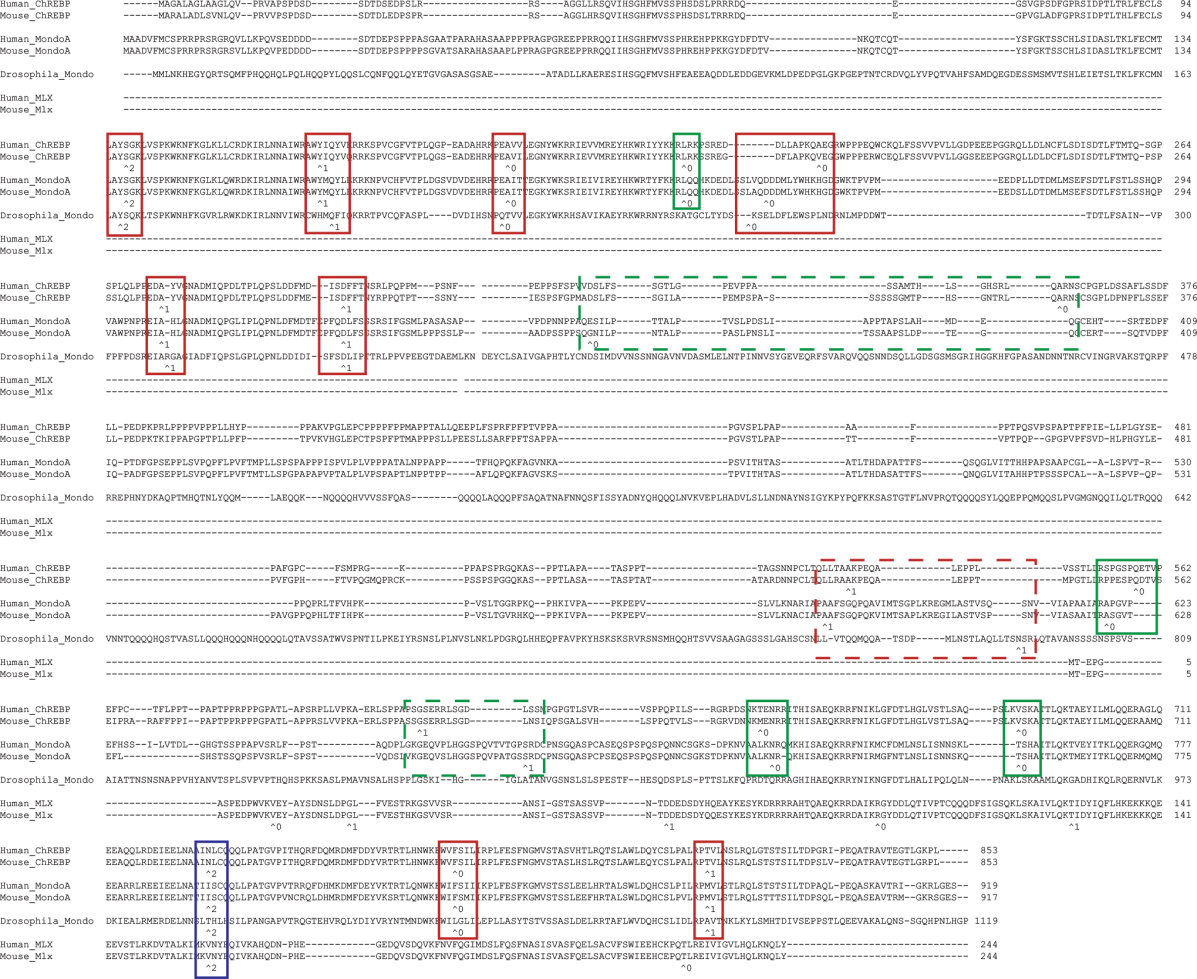
Introns in *Mlxip* and *Mlxipl* genes are at homologous locations. Alignment of human and mouse ChREBP, MondoA, and Mlx (encoded by *MLXIPL*, *MLXIP*, and *MLX*, respectively) protein sequences and *Drosophila melanogaster* Mondo (encoded by *Mio*) protein sequence with locations of introns, and phases indicated. Protein sequences were aligned with Clustal Omega. Locations of introns are indicated by ^ with the number referring to the phase of the codon interrupted by the intron. Introns at near identical locations, and of the same phase are boxed, with solid boxes indicated very similar locations, while dotted boxes are similar.

To better understand the evolution of genes, full-length gene sequences are desirable, however, identification of a full-length gene structure can be difficult, even with complete genome sequences, as gene prediction programs can misannotate genes [42,43]. Most available genomes are not complete and contain unsequenced gaps, as well as occasional sequencing errors, that increase the likelihood of obtaining incomplete gene predictions. Identification of ancestral gene structures should help identify gene structures in extant genomes. Previous studies have shown that *Mlxip* and *Mlxipl* genes are most closely related, being the products of a gene duplication early on the vertebrate lineage, with *Mlx* being their most closely related paralog [24]. Annotated human *MLXIP* (ENSG00000009950) and *MLXIPL* (ENSG00000175727) genes are both composed of 17 exons that contain coding sequence. However, *Mlxip* and *Mlxipl* genes annotated in the genomes of many vertebrate species present in the Ensembl database display a variable number of exons, raising the possibility of incorrect or incomplete annotation. To determine the structure of the ancestor of the *MLXIP* and *MLXIPL* genes, we compared the structures of well-characterized *Mlxip* and *Mlxipl* genes from human (*MLXIP*: ENSG00000175727 and *MLXIPL*: ENSG00000009950) and mouse (*Mlxip*: ENSMUSG00000038342 and *Mlxipl*: ENSMUSG00000005373), the *Drosophila melagastor* ortholog *Mio* (FBgn0032940), and their closest paralog, *Mlx* genes from human (ENSG00000108788) and mouse (ENSMUSG00000017801). The locations and phases (i.e., position within a codon interrupted by an intron [44]) of introns were mapped to an alignment of the protein sequences predicted by these genes (Fig 1). As expected, intron positions and phases were perfectly conserved between the mouse and human orthologs of the *Mlxip*, *Mlxipl*, and *Mlx* genes (Fig 1). Introns that are in similar positions in an amino acid alignment and have identical phase likely have a shared origin [44]. All *Mlxip*, *Mlxipl*, *Mio*, and *Mlx* genes shared a single intron, at codon 728 in the human *MLXIPL* gene sequence, consistent with a very ancient origin for these diverse genes (see [24]). At least 10 introns were shared between the *Mlxip*, *Mlxipl*, and *Mio* genes, with all 16 introns in the *Mlxip* and *Mlxipl* genes being located in similar positions in the amino acid alignment and having identical intron phase (Fig 1). These results strongly suggest that the ancestor of the *Mlxip* and *Mlxipl* genes was composed of 17 coding exons, separated by 16 introns, although this does not exclude the possibility of lineage specific changes in gene structure that may have occurred to some genes on specific lineages. This conclusion was also obtained for protein sequence alignments generated by other methods (which only yielded slightly differing protein sequence alignments).

### *Mlxip*, *Mlxipl*, and *Mlx* genes in vertebrates

As the ancestor of the *Mlxip* and *Mlxipl* genes was composed of 17 coding exons, we searched vertebrate genomes in the Ensembl database (www.ensembl.org) for sequences that could predict *Mlxip* and *Mlxipl* genes and contain 17 coding exons (see S1 Table and S1 and S2 Figs). Genomic sequences that predict protein sequences similar to MondoA and ChREBP identified *Mlxip* genes in all but one vertebrate species (alpaca), and *Mlxipl* genes in all but three species (tarsier, alpaca, and lamprey) (S1 Table). Similar searches for *Mlx*, the closest related paralog to *Mlxip* and *Mlxipl*, found this gene in all examined species (S1 Table and S3 Fig). These results are consistent with the previous conclusion that *Mlxip* and *Mlxipl* genes originated via genome duplication early in vertebrate evolution and that the *Mlx* gene diverged much earlier [24].

To identify intact genes, exons of the *Mlxip* (S2 Table), *Mlxipl* (S3 Table), and *Mlx* (S4 Table) genes were identified through tblastn [29] searches with the protein sequences encoded by the human *MLXIP*, *MLXIPL*, and *MLX* genes, as well as genomic alignments generated by MultiPipMaker [30,31] with the human gene sequences used as master sequences. A total of 21, 16, and 44 intact coding genes were identified for *Mlxip*, *Mlxipl*, and *Mlx*, respectively (S2, S3 and S4 Tables). The higher number of identified intact *Mlx* genes likely reflects the smaller number of exons (8 exons) in this gene compared to the *Mlxip* and *Mlxipl* genes (17 exons). In 10 mammalian species (human, chimpanzee, Vervet-African green monkey (Vervet-AGM), bushbaby, mouse, rat, guinea pig, pig, dog, and opossum) both *Mlxip* and *Mlxipl* were found to be intact (S2 and S3 Tables), and these sequences were used in the comparative analyses described below. Searches with protein sequences predicted from other intact or incomplete *Mlxip*, *Mlxipl*, and *Mlx* genes did not find any additional exons (results not shown).

### Evolution of *Mlxip* genes

While *Mlxip* genes could be found in all but one genome examined (not found in the alpaca), intact 17 coding exon genes were only identified in mammals (16 species) and fish (5 species), (S1 and S2 Tables and S1 and S4 Figs). The *Mlxip* gene was found in a single copy in all species with the gene, except the marmoset, which had two copies (S1 and S2 Tables). Of the two gene copies in the marmoset, one contained multiple substitutions (resulting in frameshifts or inframe stop codons in 6 exons) incompatible with function indicating that it was a pseudogene (S1 and S2 Tables). None of the *Mlxip* genes found in any other vertebrate species presented strong evidence of being a pseudogene (i.e., possessing multiple mutations that disrupt the open reading frame), although a few mutations that introduce stop codons or frameshifts were found in some genes (e.g., tree shrew and kangaroo rat, see S2 Table), however all of these were in incomplete genes and often found in genomes with low coverage and thus may simply be sequencing errors. Only a single copy *Mlxip* gene was found in each of the fish genomes examined, despite these species experiencing a genome duplication [45]. Phylogenetic analysis of the 21 intact *Mlxip* coding sequences by maximum likelihood, Bayesian, and neighbor-joining methods yielded a phylogeny consistent with the accepted species phylogeny (S5 Fig), with no strong evidence for lineage-specific changes in rates of evolution, suggesting no major changes in the function (and thus selective constraints) of these proteins in the examined species.

### Evolution of *Mlxipl* genes

Like *Mlxip*, *Mlxipl* genes were found in almost all vertebrates (not found in only alpaca, tarsier and lamprey), with intact 17 coding exon genes found in mammals (14 species), birds (1 species), and a reptile (1 species), but not in amphibians or fish (S1 and S3 Tables and S2 and S6 Figs). The full-length chicken and rat coding sequences were obtained with sequences from the NCBI database, as the genomic sequence found in the Ensembl database was incomplete (see S3 Table). No evidence for a duplicate *Mlxipl* gene was found in any species. Again, mutations that introduce stop codons or frameshifts were found in some incomplete *Mlxipl* genes (e.g., tree shrew and kangaroo rat, see S2 Table), however these were found in genomes with low coverage and may simply be sequencing errors. The opossum *Mlxipl* gene possessed a frameshift mutation in an otherwise intact coding sequence (see S3 Table), but this mutation was in a portion of the genomic sequence that was of low quality (only three poor quality shotgun sequence reads overlapped this region), thus we considered this to be a sequencing error. Like *Mlxip*, only a single copy of the *Mlxipl* gene was found in fish genomes. Phylogenetic analysis of the 16 intact *Mlxipl* coding sequences by both maximum likelihood and Bayesian methods yielded a phylogeny consistent with the accepted species phylogeny (S7 Fig) with no strong evidence for lineage-specific variations in rates of evolution, thus also suggesting that among the examined ChREBP protein sequences no change in function (or selective constraint) had occurred.

Alternative promoters, and adjacent first exons, have been described for mammalian *Mlxipl* genes in a few mammalian species, which generate two different ChREBP isoforms [20,22]. The alpha isoform (ChREBP-α) corresponds the full-length sequence, while the beta isoform (ChREBP-β) is generated using an alternative promoter with alternative splicing that skips exon 1 producing a transcript that allows translation to initiate at a downstream ATG codon in exon 4 [22]. Examination of our predicted ChREBP protein sequences, including partial sequences, shows that the ChREBP-β specific downstream ATG codon in exon 4 is perfectly conserved in mammals, birds, reptiles, and amphibians, but not in bony (e.g., zebrafish and spotted gar) or lobe-finned (i.e., coelacanth) fish where instead this codon codes for isoleucine. No conserved inframe ATG was found in fish, either upstream or downstream of the ChREBP-β isoform ATG codon, which could have suggested a different N-terminus for the β isoform, indicating that the ChREBP-β isoform is tetrapod specific and evolved in the common ancestor of tetrapods after the divergence of this lineage from the lobe-finned fish lineage. Unfortunately, due to the failure to identify intact 17 exon *Mlxipl* genes in fish, we cannot determine whether changes in the evolutionary constraints (potentially due to a change in protein function) occurred with the origin of the ChREBP-β isoform, although one might expect this to have occurred.

### Evolution of *Mlx* genes

In contrast to *Mlxip* and *Mlxipl*, *Mlx* genes were found in the genomes of all species examined (S1 and S4 Tables and S3 and S8 Figs). A larger number of intact *Mlx* genes were identified, which may not be unexpected as the gene contains only 8 coding exons. Again, a few mutations (in alpaca and hedgehog, see S4 Table) were identified that disrupt coding potential, but are possibly sequencing errors as they are found in lower quality genomes. Phylogenetic analysis of the 44 intact *Mlx* coding sequences by both maximum likelihood and Bayesian methods yielded a phylogeny consistent with the accepted species phylogeny (S9 Fig). In contrast to *Mlxip* and *Mlxipl*, a duplicated *Mlx* gene was found in a fish genome, however, the phylogenetic analysis (S9 Fig) indicates that this is a very recent duplication and not a product of the fish-specific genome duplication. Thus, despite an opportunity for sub-functionalization after the fish-specific duplication, the retention of a single *Mlx* gene suggests that there is strong selection to maintain coordinated interaction between Mlx and both ChREBP and MondoA. Since Mlx interacts with a large number of other basic-helix-loop-helix proteins [19,24], it is not surprising that no evidence for changes in selective constraints acting on this gene was seen in the phylogenetic analysis (S9 Fig).

### Differences in the rates of evolution of *Mlxip* and *Mlxipl* coding sequences

**Fig 2:**
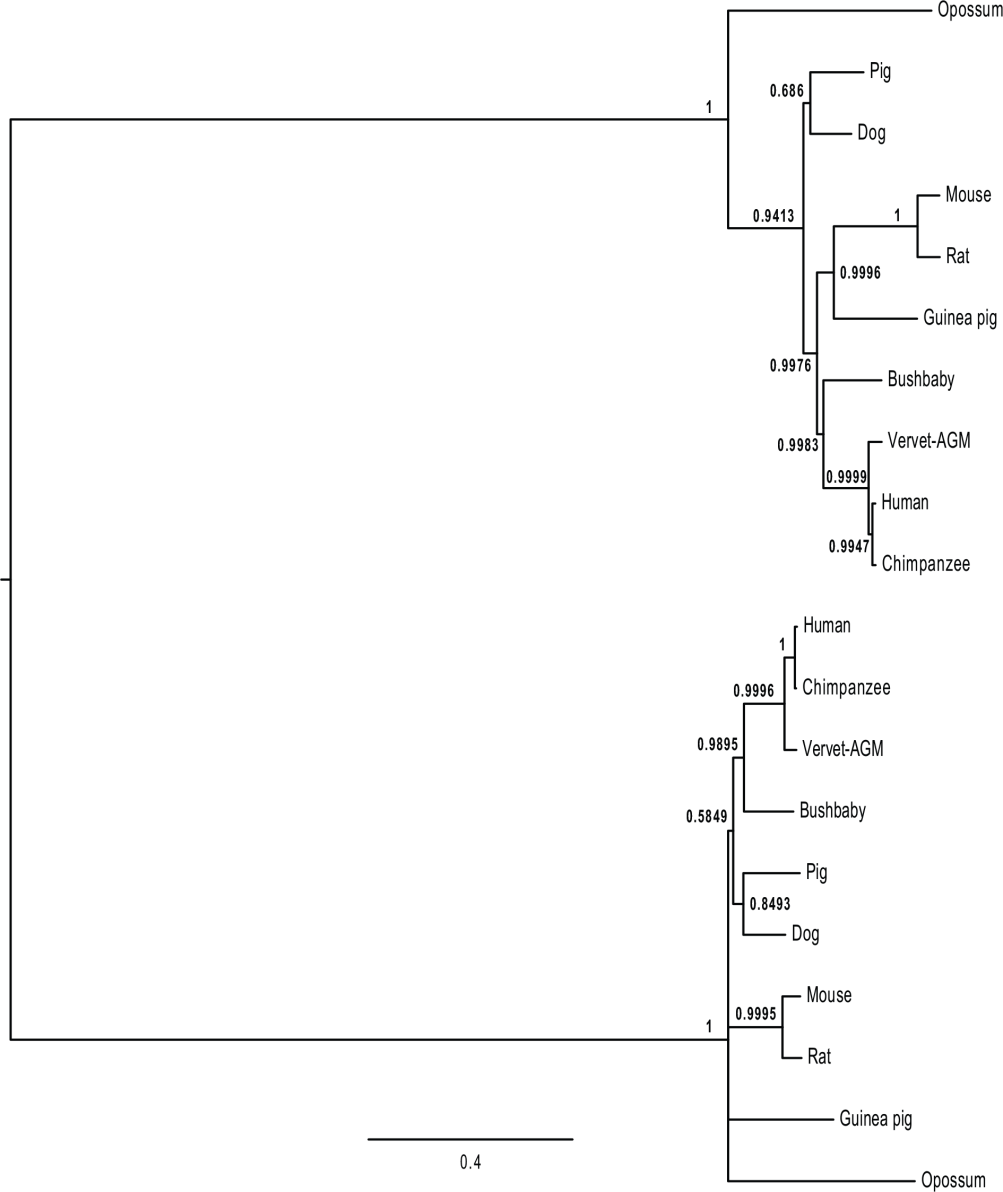
Phylogeny of *Mlxip* and *Mlxipl* coding sequences. Phylogeny inferred by the Bayesian method, implemented in MrBayes version 3.2.2, is shown, using the coding sequence alignment from S2 Fig based on the alignment presented in S12 Fig. The phylogeny is rooted between the sequences for *Mlxip* (shown in the upper portion) and *Mlxipl* (lower portion). Branch lengths are proportional to the number of inferred nucleotide substitutions. Numbers at the node represent posterior probabilities after 2,000,000 generations. Similar phylogenies were generated when Maximum likelihood or neighbor-joining methods were used.

Both MondoA and ChREBP interact with Mlx to form transcription factors that regulate distinct sets of genes [15,19,25]. As such, it would be expected that the selective forces acting on these two genes should differ. To identify sequences within the MondoA and ChREBP proteins that may evolve in different patterns, we examined the evolution of the *Mlxip* and *Mlxipl* genes that encoded full-length coding sequences. To prevent lineage-specific effects, we only examined genes from species that had intact coding sequences for both *Mlxip* and *Mlxipl* genes, thus evolved in parallel in the same genomic environments and the differences in evolutionary patterns detected should reflect the consequences of selection for gene-specific functions. Within mammals, a total of 10 species were found to contain intact copies of the *Mlxip* and *Mlxipl* genes (S2 and S3 Tables). These sequences were used to compare and contrast the evolution of *Mlxip* and *Mlxipl* as they represent the diversity of mammals and their lineages contain equal amounts of evolutionary time and, as they have coexisted in the same genome on all lineages, should have experienced identical evolutionary pressures at the genome level. An alignment of the protein sequences is shown in S10 and S11 Figs, where intron positions are identical within genes and at very similar location in the alignment between genes (similar alignments were generated using other multiple sequence aligners). The phylogeny (Fig 2) of these genes is consistent with phylogenies of the species (with very similar phylogenies generated by other methods), with the gene-specific phylogenies consistent with those from large numbers of genes (S2 and S3 Figs).

**Fig 3:**
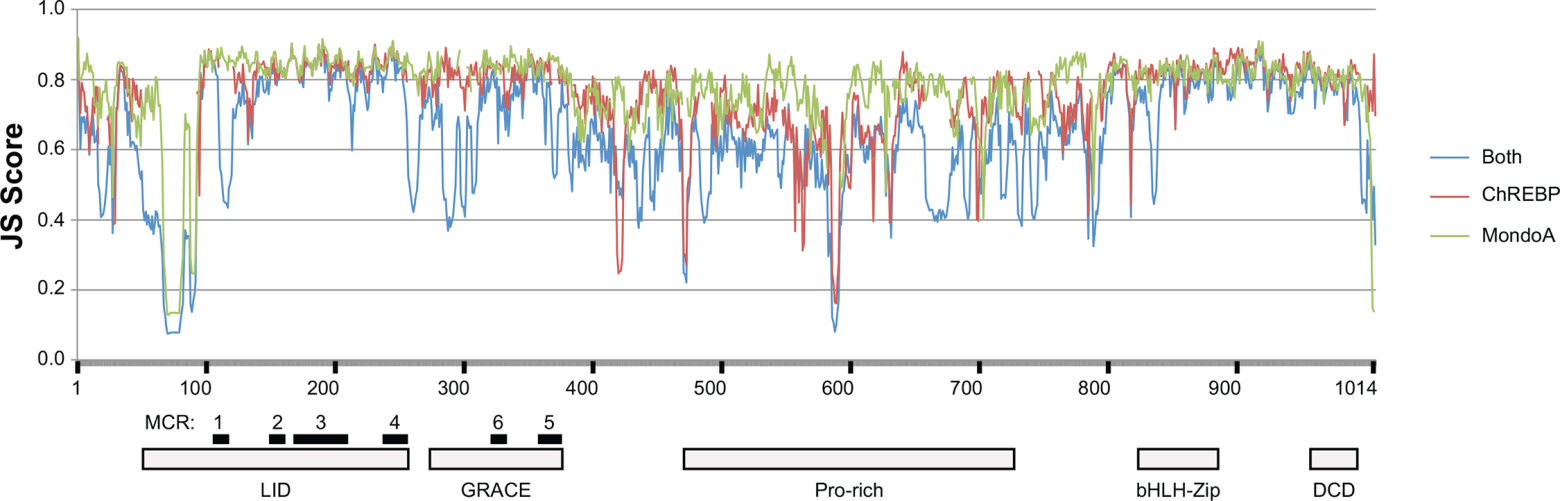
Variability in MondoA and ChREBP protein sequences. JS Divergence scores across the alignment of MondoA and ChREBP protein sequences. Plots of JS Scores for MondoA, ChREBP and combined MondoA and ChREBP protein sequences are shown. JS Scores for each position are presented in S5 Table. A schematic organization of the domains in MondoA and ChREBP is shown below the plot. Alignment of sequences and locations of domains are shown in S7 Fig.

To identify portions of the MondoA and ChREBP protein sequences that are evolving in different patterns we compared the conservation of the sequences across the lengths of these proteins. Jenson-Shannon Divergence (JS) scores, which measure conservation over a window of sites [40], were used to assess the conservation of the sequences across the entire protein sequence (S5 Table), with the results displayed in Fig 3. As expected, the previously identified functional domains typically show higher JS scores, especially the Mondo Conserved Regions (MCR1-6) and the basic-helix-loop-helix leucine-zipper (bHLH-Zip) and dimerization and cytoplasmic localization (DCD) domains (Fig 3). Despite equal amounts of evolutionary time being represented by the MondoA and ChREBP sequences, some differences in the JS divergence scores can be seen across the protein length (Fig 3). To quantify differences in the conservation in the domains of the MondoA and ChREBP proteins we averaged the JS Divergence Scores for each domain (Table 1).

**Table 1:** Comparison of the average JS Divergence scores for domains in MondoA and ChREBP protein sequences.

Domain^1^	ChREBP	MondoA	Both^2^	Length^3^
All	**0.76541**^4^	0.79164	0.70455	824
LID	**0.82599**	0.84046	0.79199	157
MCR1	**0.84506**	0.85847	0.82470	15
MCR2	**0.82402**	0.83990	0.80249	13
MCR3	**0.84459**	0.85337	0.83460	44
MCR4	**0.84333**	0.84849	0.81087	40
GRACE	**0.81397**	0.83814	0.75050	80
MCR6	**0.83503**	0.84107	0.77072	12
MCR5	**0.83818**	0.84010	0.79425	21
Pro-rich	**0.67853**	0.74434	0.60883	209
bHLH-Zip	**0.80920**	0.82152	0.76753	61
DCD	0.81806	**0.80635**	0.77976	41

^1^ Domains are from S4 Fig.

^2^ Both are scores from the alignment of MondoA and ChREBP sequences.

^3^ Length of compared region, average scores were calculated after deletion of all positions that had gaps in any sequence.

^4^ Scores in bold indicate lower levels of sequence conservation.

**Fig 4:**
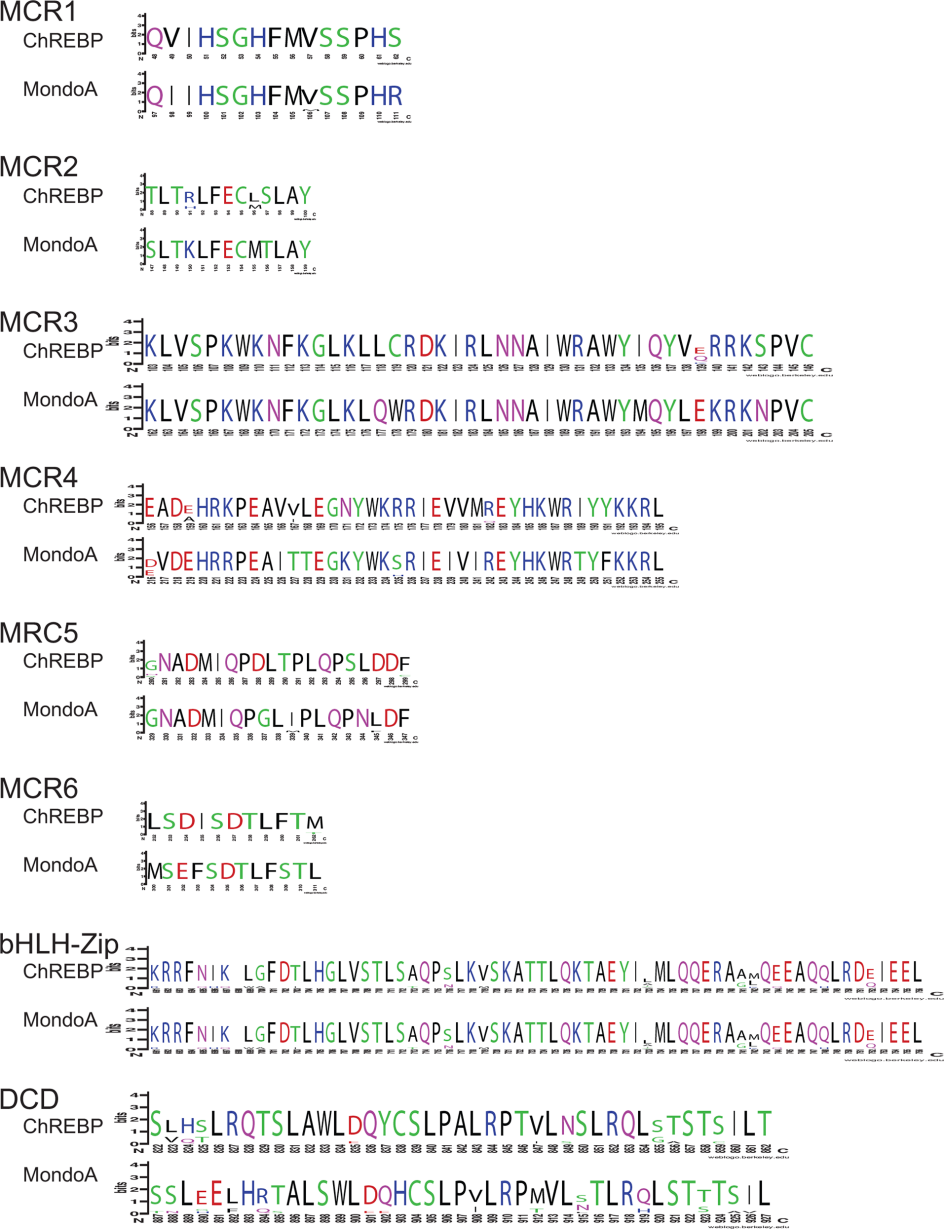
Consensus sequences for MCR1-6, bHLH-Zip and DCD domains in mammalian MondoA and ChREBP protein sequences. Consensus sequences from 10 mammalian ChREBP and MondoA protein sequences of Mondo Conserved Regions (MCR1-6), basic helix-loop helix leucine-zipper (bHLH-Zip) and the dimerization and cytoplasmic localization (DCD) domains are displayed by WebLogos. Numbers below residues indicate position in alignments of the 10 mammalian ChREBP or MondoA sequences. Heights of letters indicate abundance of that residue in the 10 sequences. Color of residues indicate chemical properties, with green being polar amino acids (G, S, T, Y, C, Q, and N), blue are basic (K, R, and H), red are acidic (D and E) and black are hydrophobic (A, V, L, I, P, W, F, and M).

When the divergence of the different domains in the MondoA and ChREBP protein sequences are compared, the LID, GRACE, Proline-rich, and bHLH-Zip domains the ChREBP sequences show lower levels of sequence conservation, while the MondoA proteins show lower conservation levels only for the DCD domain (Table 1). A similar pattern is observed if pairwise observed sequence difference is examined, where the majority of the pairwise differences for ChREBP sequence are greater than those for MondoA sequences for the LID, GRACE, Proline-rich, and bHLH-Zip domains, but lower for the DCD domain (S6 Table). To examine more closely the effects of the differing levels of constraints we focused on the consensus sequences for the Mondo Conserved Regions (MCR1-6), bHLH-Zip, and DCD domains, portions of the sequences that have functional roles [15,20,26–28]. Consensus sequences for the domains in ChREBP and MondoA from the 10 mammals were calculated using WebLogo [41] and shown in Fig 4.

As shown in Fig 4, the majority of differences between the consensus sequences in ChREBP and MondoA involve replacements of residues with those of similar chemical properties (e.g., position 2 of MCR1 and position 4 of MCR2), however some more radical substitutions were detected (e.g., positions 16 and 17 of MCR3, position 20 of MCR4, and position 9 of MCR5). Variations in the length of MCR5 and MCR6 were detected between ChREBP and MondoA, with MCR5 being one residue longer in ChREBP and MCR6 being one longer in MondoA (Fig 4). MCR1 and MCR2 displayed very similar sequences, with the only differences being those that retain chemical properties, suggesting that they function similarly. Greater levels of variation are seen in the consensus sequences for the remaining MCRs (MCR3-6), with the ChREBP sequences generally showing lower levels of conservation (also seen in Table 1), suggests that differences in function and constraints exist between these sequences, potentially due to interacting with a smaller number of proteins. These patterns suggest that stronger selection acted on the MondoA sequence than on ChREBP, implying that MondoA is more crucial for survival. Differences in the consensus sequences for the bHLH-Zip and DCD domains likely yield differences in binding site preferences and the regulation of downstream genes. Intriguingly, the bHLH-Zip shows less constraint in ChREBP, while the DCD domain is more variable in MondoA (Table 1). Thus it appears that changes in their downstream target genes and pathways have been acquired by mutations occurring in parallel in these two different domains of the proteins.

## Conclusions

The *Mlxipl* and *Mlxip* genes encoding the glucose-responsive transcription factors ChREBP and MondoA are found in single copy in almost all vertebrates, as is the gene *Mlx* encoding their required interacting partner. These observations support the previous conclusion that *Mlxipl* and *Mlxip* are products of the genome duplication on the very early vertebrate lineage, and that *Mlx* has a more ancient relationship [24]. The failure to find additional duplicated *Mlxipl*, *Mlxipl*, or *Mlx* genes in many species, especially fish that experienced a genome duplication [45] suggests that duplication of any of these genes is likely disruptive. The single exception is the duplicated *Mlx* genes in the Amazon molly (see S1 Table and S6 Fig), two genes that are nearly identical in sequence. Selection may not have had enough time to act on this very recent gene duplication. Both ChREBP and MondoA are glucose-responsive, however, they regulate different sets of downstream genes, with ChREBP largely responsible for regulating lipogenesis in the liver while MondoA regulates the glycolytic pathway in muscle cells [15,19]. Given the differences in the functions of ChREBP and MondoA, it might be unexpected that subfunctionalization of Mlx function, to specialize paralogs for interaction with ChREBP or MondoA has not occurred. The failure to subfunctionalize Mlx function might indicate that natural selection favors the retention of a single interacting partner for both ChREBP and MondoA. Mlx interacts with not only ChREBP and MondoA, but also other members of the basic helix-loop-helix leucine-zipper transcription family [19,24]. Possession of a single *Mlx* gene thus potentially allows coordination of a series of transcription factors.

Since ChREBP and MondoA are both glucose-sensitive transcription factors that regulate genes that control complementary aspects of energy metabolism, it is expected that the selective constraints acting upon these related sequences should differ. Phylogenetic analysis of intact *Mlxip* and *Mlxipl* coding sequences did not suggest any lineage-specific variation in the rates of evolution (S1 and S2 Figs), suggesting that each gene has evolved at more-or-less uniform rates within mammals, however given the low number of non-mammalian sequences it is possible that variation in rates of evolution occur among classes of vertebrates. To compare rates between genes, we used a set of 10 mammals that contain intact copies of both the *Mlxip* and *Mlxipl* genes (Fig 2). Using this set of 10 species, we can compare the levels of selection acting across the sequences of both genes over a long evolutionary history where both genes shared identical phylogenetic and genomic history. ChREBP was found to be generally less conserved and show greater divergence than MondoA across most of the sequence, although it showed greater conservation of the dimerization and cytoplasmic localization (DCD) domain (Figs 3 and 4, Table 1, and S6 Table). This result suggests that the MondoA sequence is under greater evolutionary constraint then ChREBP, potentially due to interactions with a greater number of other proteins or genes. Comparison of ChREBP protein sequences demonstrated that the N-terminus of the ChREBP-β isoform evolved with the origin of tetrapods. Since the ChREBP-β isoform appears to be key to regulating ChREBP function [22,23], this may suggest that ChREBP function is more recently evolved, with MondoA performing more ancestral, and potentially more evolutionary important, function.

## Supporting Information

S1 Fig *Mlxip* coding sequences. (TXT)

S2 Fig *Mlxipl* coding sequences. (TXT)

S3 Fig *Mlx* coding sequences. (TXT)

S4 Fig Fasta formatted alignment of intact *Mlxip* coding sequences. (TXT)

S5 Fig Phylogeny of intact *Mlxip* genes. (EPS)

S6 Fig Fasta formatted alignment of intact *Mlxipl* coding sequences. (TXT)

S7 Fig Phylogeny of intact *Mlxipl* genes. (EPS)

S8 Fig Fasta formatted alignment of intact *Mlx* coding sequences. (TXT)

S9 Fig Phylogeny of intact *Mlx* genes. (EPS)

S10 Fig Alignment of ChREBP and MondoA sequences from species that contain complete coding sequences for both proteins. (DOCX)

S11 Fig Fasta formatted alignment of MondoA and ChREBP protein sequences from species that contain both complete coding sequences. (TXT)

S12 Fig Fasta formatted alignment of *Mlxip* (MondoA) and *Mlxipl* (ChREBP) coding sequences from species that contain both complete coding sequences. (TXT)

S1 Table Locations of *Mlx*, *Mlxip*, and *Mlxipl* genes in vertebrate genomes. (XLSX)

S2 Table Presence or absence of exons in identified *Mlxip* genes. (XLSX)

S3 Table Presence or absence of exons in identified *Mlxipl* genes. (XLSX)

S4 Table Presence or absence of exons in identified Mlx genes. (XLSX)

S5 Table JS divergence scores for mammalian *Mlxip* and *Mlxipl* coding sequences. (XLSX)

S6 Table Observed pairwise differences in MondoA and ChREBP protein and domain sequences. (XLS)

## References

[1] Moore MC, Coate KC, Winnick JJ, An Z, Cherrington AD. Regulation of hepatic glucose uptake and storage in vivo. Adv Nutr. 2012; 3: 286–294. 10.3945/an.112.002089 22585902 PMC3649460

[2] Rui L. Energy metabolism in the liver. Compr Physiol. 2014; 4:177–197. 10.1002/cphy.c130024 24692138 PMC4050641

[3] Rojas JM, Schwartz MW. Control of hepatic glucose metabolism by islet and brain. Diabetes Obes Metab. 2014; 16 Suppl 1: 33–40. 10.1111/dom.12332 25200294 PMC4191916

[4] Agius L. Glucokinase and molecular aspects of liver glycogen metabolism. Biochem J. 2008; 414: 1–18. 10.1042/BJ20080595 18651836

[5] Panserat S, Rideau N, Polakof S. Nutritional regulation of glucokinase: a cross-species story. Nutr Res Rev. 2014; 27: 21–47. 10.1017/S0954422414000018 24896238

[6] Thorens B. GLUT2, glucose sensing and glucose homeostasis. Diabetologia. 2015; 58: 221–232. 10.1007/s00125-014-3451-1 25421524

[7] Oh KJ, Han HS, Kim MJ, Koo SH. CREB and FoxO1: two transcription factors for the regulation of hepatic gluconeogenesis. BMB Rep. 2013; 46: 567–574. 24238363 10.5483/BMBRep.2013.46.12.248 PMC4133859

[8] Oosterveer MH, Schoonjans K. Hepatic glucose sensing and integrative pathways in the liver. Cell Mol Life Sci. 2014; 71: 1453–1467. 10.1007/s00018-013-1505-z 24196749 PMC11114046

[9] Wasserman DH. Four grams of glucose. Am J Physiol Endocrinol Metab. 2009; 296: E11–21. 10.1152/ajpendo.90563.2008 18840763 PMC2636990

[10] Iynedjian PB. Molecular physiology of mammalian glucokinase. Cell Mol Life Sci. 2009; 66: 27–42. 10.1007/s00018-008-8322-9 18726182 PMC2780631

[11] Hirota K, Fukamizu A. Transcriptional regulation of energy metabolism in the liver. J Recept Signal Transduct Res. 2010; 30: 403–409. 10.3109/10799893.2010.509730 20735177

[12] Kahn A. Transcriptional regulation by glucose in the liver. Biochimie. 1997; 79: 113–118. 9209706 10.1016/s0300-9084(97)81501-5

[13] Yamashita H, Takenoshita M, Sakurai M, Bruick RK, Henzel WJ, Shillinglaw W, Arnot D, Uyeda K. A glucose-responsive transcription factor that regulates carbohydrate metabolism in the liver. Proc Natl Acad Sci U S A. 2001; 98: 9116–9121. 11470916 10.1073/pnas.161284298 PMC55382

[14] Uyeda K, Yamashita H, Kawaguchi T. Carbohydrate responsive element- binding protein (ChREBP): a key regulator of glucose metabolism and fat storage. Biochem Pharmacol. 2002; 63: 2075–2080. 12110366 10.1016/s0006-2952(02)01012-2

[15] Havula E, Hietakangas V. Glucose sensing by ChREBP/MondoA-Mlx transcription factors. Semin Cell Dev Biol. 2012; 23: 640–647. 10.1016/j.semcdb.2012.02.007 22406740

[16] de Luis O, Valero MC, Jurado LA. WBSCR14, a putative transcription factor gene deleted in Williams-Beuren syndrome: complete characterization of the human gene and the mouse ortholog. Eur J Hum Genet. 2000; 8: 215–222. 10780788 10.1038/sj.ejhg.5200435

[17] Cairo S, Merla G, Urbinati F, Ballabio A, Reymond A. WBSCR14, a gene mapping to the Williams—Beuren syndrome deleted region, is a new member of the Mlx transcription factor network. Hum Mol Genet. 2001; 10: 617–627. 11230181 10.1093/hmg/10.6.617

[18] Stoeckman AK, Ma L, Towle HC. Mlx is the functional heteromeric partner of the carbohydrate response element-binding protein in glucose regulation of lipogenic enzyme genes. J Biol Chem. 2004; 279: 15662–15669. 14742444 10.1074/jbc.M311301200

[19] Billin AN, Eilers AL, Queva C, Ayer DE. Mlx, a novel Max-like BHLHZip protein that interacts with the Max network of transcription factors. J Biol Chem. 1999; 274: 36344–36350. 10593926 10.1074/jbc.274.51.36344

[20] Iizuka K. Recent progress on the role of ChREBP in glucose and lipid metabolism. Endocr J. 2013; 60: 543–555. 23604004 10.1507/endocrj.ej13-0121

[21] Li MV, Chang B, Imamura M, Poungvarin N, Chan L. Glucose-dependent transcriptional regulation by an evolutionarily conserved glucose-sensing module. Diabetes. 2006; 55: 1179–1189. 16644671 10.2337/db05-0822

[22] Herman MA, Peroni OD, Villoria J, Schön MR, Abumrad NA, Blüher M, Klein S, Kahn BB. A novel ChREBP isoform in adipose tissue regulates systemic glucose metabolism. Nature. 2012; 484: 333–338. 10.1038/nature10986 22466288 PMC3341994

[23] Eissing L, Scherer T, Tödter K, Knippschild U, Greve JW, Buurman WA, Pinnschmidt HO, Rensen SS, Wolf AM, Bartelt A, Heeren J, Buettner C, Scheja L. De novo lipogenesis in human fat and liver is linked to ChREBP-β and metabolic health. Nat Commun. 2013; 4: 1528 10.1038/ncomms2537 23443556 PMC3740744

[24] McFerrin LG, Atchley WR. Evolution of the Max and Mlx networks in animals. Genome Biol Evol. 2011; 3: 915–937. 10.1093/gbe/evr082 21859806 PMC3177325

[25] Ma L, Tsatsos NG, Towle HC. Direct role of ChREBP.Mlx in regulating hepatic glucose-responsive genes. J Biol Chem. 2005; 280: 12019–12027. 15664996 10.1074/jbc.M413063200

[26] Eilers AL, Sundwall E, Lin M, Sullivan AA, Ayer DE. A novel heterodimerization domain, CRM1, and 14-3-3 control subcellular localization of the MondoA-Mlx heterocomplex. Mol Cell Biol. 2002; 22: 8514–8526. 12446771 10.1128/MCB.22.24.8514-8526.2002 PMC139889

[27] McFerrin LG, Atchley WR. A novel N-terminal domain may dictate the glucose response of Mondo proteins. PLoS One. 2012; 7: e34803 10.1371/journal.pone.0034803 22506051 PMC3323566

[28] Eilers AL, Sundwall E, Lin M, Sullivan AA, Ayer DE. A novel heterodimerization domain, CRM1, and 14-3-3 control subcellular localization of the MondoA-Mlx heterocomplex. Mol Cell Biol. 22: 8514–8526. 12446771 10.1128/MCB.22.24.8514-8526.2002 PMC139889

[29] Gertz EM, Yu YK, Agarwala R, Schäffer AA, Altschul SF. Composition-based statistics and translated nucleotide searches: improving the TBLASTN module of BLAST. BMC Biol. 2006; 4: 41 17156431 10.1186/1741-7007-4-41 PMC1779365

[30] Schwartz S, Zhang Z, Frazer KA, Smit A, Riemer C, Bouck J, et al PipMaker—a web server for aligning two genomic DNA sequences. Genome Res. 2000; 10: 577–586. 10779500 10.1101/gr.10.4.577 PMC310868

[31] Schwartz S, Elnitski L, Li M, Weirauch M, Riemer C, Smit A, et al MultiPipMaker and supporting tools: Alignments and analysis of multiple genomic DNA sequences. Nucleic Acids Res. 2003; 31: 3518–3524. 12824357 10.1093/nar/gkg579 PMC168985

[32] Smit AFA, Hubley R, Green P. RepeatMasker Open-3.0. 1996–2010. Available:http://www.repeatmasker.org.

[33] Sievers F, Wilm A, Dineen D, Gibson TJ, Karplus K, Li W, Lopez R, McWilliam H, Remmert M, Söding J, Thompson JD, Higgins DG. Fast, scalable generation of high-quality protein multiple sequence alignments using Clustal Omega. Mol Syst Biol. 2011; 7: 539 10.1038/msb.2011.75 21988835 PMC3261699

[34] Katoh K, Misawa K, Kuma K, Miyata T. MAFFT: a novel method for rapid multiple sequence alignment based on fast Fourier transform. Nucl Acids Res. 2002; 30: 3059–3066. 12136088 10.1093/nar/gkf436 PMC135756

[35] Penn O, Privman E, Ashkenazy H, Landan G, Graur D, Pupko T. GUIDANCE: a web server for assessing alignment confidence scores. Nucl Acids Res. 2010; 38: W23–W28. 10.1093/nar/gkq443 20497997 PMC2896199

[36] Ronquist F, Teslenko M, van der Mark P, Ayres DL, Darling A, Höhna S, et al MrBayes 3.2: efficient Bayesian phylogenetic inference and model choice across a large model space. Syst Biol. 2012; 61: 539–542. 10.1093/sysbio/sys029 22357727 PMC3329765

[37] Guindon S, Dufayard JF, Lefort V, Anisimova M, Hordijk W, Gascuel O. New algorithms and methods to estimate maximum-likelihood phylogenies: assessing the performance of PhyML 3.0. Syst Biol. 2010; 59: 307–321. 10.1093/sysbio/syq010 20525638

[38] Tamura K, Stecher G, Peterson D, Filipski A, Kumar S. MEGA6: Molecular Evolutionary Genetics Analysis Version 6.0. Mol Biol Evol. 2013; 30: 2725–2729. 10.1093/molbev/mst197 24132122 PMC3840312

[39] Posada D, Crandall KA. Selecting the best-fit model of nucleotide substitution. Syst Biol. 2001; 50: 580–601. 12116655

[40] Capra JA, Singh M. Predicting functionally important residues from sequence conservation. Bioinformatics. 2007; 23: 1875–1882. 17519246 10.1093/bioinformatics/btm270

[41] Crooks GE, Hon G, Chandonia JM, Brenner SE. WebLogo: a sequence logo generator. Genome Res. 2004; 14: 1188–1190. 15173120 10.1101/gr.849004 PMC419797

[42] Harrow J, Nagy A, Reymond A, Alioto T, Patthy L, Antonarakis SE, Guigó R. Identifying protein-coding genes in genomic sequences. Genome Biol. 2009; 10: 201 10.1186/gb-2009-10-1-201 19226436 PMC2687780

[43] Yandell M, Ence D. A beginner's guide to eukaryotic genome annotation. Nat Rev Genet. 2012; 13: 329–342. 10.1038/nrg3174 22510764

[44] Patthy L. Intron-dependent evolution: preferred types of exons and introns. FEBS Lett. 1987; 214: 1–7. 3552723 10.1016/0014-5793(87)80002-9

[45] Meyer A, Van de Peer Y. From 2R to 3R: evidence for a fish-specific genome duplication (FSGD). Bioessays. 2005; 27: 937–945. 16108068 10.1002/bies.20293

