# Supplementary material for: Contrasting Patterns in the Evolution of Vertebrate MLX Interacting Protein (MLXIP) and MLX Interacting Protein-Like (MLXIPL) Genes
Source: PLoS One. 2016 Feb 24;11(2):e0149682. doi: 10.1371/journal.pone.0149682 (PMC4766361; doi:10.1371/journal.pone.0149682)
Supplement: S10 Fig — (DOCX) [file pone.0149682.s010.docx]

>>> MCR1 <<< >>> MCR2 <<< >>> MCR3

>>>> LID

Human_CHREBP MAGALAGLAAGLQVPRV------APSP---DSDSDTDSEDPSLRRSAGGLL------------------------------------------RS-QVIHSGHFMVSSPHS----------DSLPRRRDQEGSVGPSDFGPRSIDPTLTRLFECLSLAYSGKLVSPKWKNFKGLKLLCRD

Chimpanzee_ChREBP MAGALAGLAAGLQVPRV------APSP---DSDSDTDSEDPSLRRSAGGLL------------------------------------------RS-QVIHSGHFMVSSPHS----------DSLPRRRDQEGSVGPSDFGPRSIDPTLTRLFECLSLAYSGKLVSPKWKNFKGLKLLCRD

Vervet-AGM_ChREBP MAGALAGLAAGLQVPRV------APSP---DSDSDTDSEDPSLRRSASCLL------------------------------------------RS-QVIHSGHFMVSSPHS----------DSLPRRRDQEGSLGPSDFGPRSIDPTLTRLFECLSLAYSGKLVSPKWKNFKGLKLLCRD

Pig_ChREBP MAGALAGLVAGLQGPRL------VPSP---DSDSDTDSEDPSTRRSAGGLL------------------------------------------RS-QVIHSGHFMVSSPHS----------DSLTRRRDQEGPLGLADFGPRSIDPTLTRLFECMSLAYSGKLVSPKWKNFKGLKLLCRD

Dog_ChREBP MAGALAGLAAGLHGPRG------VPSQ---DSDSDTDSEDPSARRSAGGLL------------------------------------------RS-QVIHSGHFMVSSPHS----------DSLPRRRDQEGPMGPADFGPRSIDPTLTRLFECMSLAYSGKLVSPKWKNFKGLKLLCRD

Bushbaby_ChREBP MAGALAGLATGLQVPRI------APSP-DSDSDSDTDSEDPNLRRSAGGLL------------------------------------------RS-QVIHSGHFMVSSPHS----------DSLTRRRDQEGPGGLADFGPRSIDPTLTRLFECLSLAYSGKLVSPKWKNFKGLKLLCRD

Mouse_ChREBP MARALADLSVNLQVPRV------VPSP---DSDSDTDLEDPSPRRSAGGLH------------------------------------------RS-QVIHSGHFMVSSPHS----------DSLTRRRDQEGPVGLADFGPRSIDPTLTHLFECLSLAYSGKLVSPKWKNFKGLKLLCRD

Rat_ChREBP MARALADLSVNLQVPRV------VPSP---DSDSDTDLEDPSPRRSAGGLH------------------------------------------RS-QVIHSGHFMVSSPHS----------DSLTRRRDQEGPVGLADFGPRSIDPTLTRLFECLSLAYSGKLVSPKWKNFKGLKLLCRD

Guinea_pig_ChREBP MARALAGLASRLQVPRV------VSSP---DSDSDTDSEDPGPRRSAGGLL------------------------------------------RS-QVIHSGHFMVSSPHS----------DSLTRRRDQEGPIGLGDFGPRSIDPTLTHLFECLSLAYSGKLVSPKWKNFKGLKLLCRD

Opossum_ChREBP MAGALAGLAGGFPGPRA------EPAP---DSDSDTDSEGPGPRRGASSLL------------------------------------------RSPQVIHSGHFMVSSPHS----------DSVPRRGAQEGPVGPGDFGPRSIDPTLTRLFECMSLAYSGKLVSPKWKNFKGLKLLCRD

^2

Human_MONDOA MAADVFMCSPRRPRSRGRQVLLKPQVS-EDDDDSDTDEPSPPPASGAATPARAHASAAPPPPRAGPG----------------REEPP-----RRQQIIHSGHFMVSSPHREHPPKKGYDFDTVNKQTCQTYSFGKTSSCHLSIDASLTKLFECMTLAYSGKLVSPKWKNFKGLKLQWRD

Chimpanzee_MondoA MAADVFMCSPRRPRSRGRQVLLKPQVS-EDDDDSDTDEPSPPPASGAATPARAHASAAPPPPRAGPG----------------REEPP-----RRQQIIHSGHFMVSSPHREHPPKKGYDFDTVNKQTCQTYSFGKTSSCHLSIDASLTKLFECMTLAYSGKLVSPKWKNFKGLKLQWRD

Vervet-AGM_MondoA MAADVFMCSPRRPRSRGRQVLLKPQVP-EDDDDSDTDEPSPPPASGAATPARAHASAAPPPPRAGPG----------------REEPP-----RRQQIIHSGHFMVSSPHREHPPKKGYDFDTVNKQTCQTYSFGKTSSCHLSIDASLTKLFECMTLAYSGKLVSPKWKNFKGLKLQWRD

Bushbaby_MondoA MAADVFMCSPRRPGNRGRPVLLKPQVP-EDDDDSDTDEPSPPPASGATAAARAHASAAQHPPRAGPG----------------REEPP-----RRQQIIHSGHFMVSSPHREHPPKKGYDFDTVNKQTCQTYSFGKTSSCHLSIDASLTKLFECMTLAYSGKLVSPKWKNFKGLKLQWRD

Pig_MondoA MAAEVFLCSGRRPHSRGRAVLLKPQVP-EDDDDSDTDEPAPPPASGAATLARGHASAGPPQPRAGSG----------------REEPP-----RRQQIIHSGHFMVSSPHREHPPKKGYDFDTVNKQTCQTYSFGKTSSCHLSIDASLTKLFECMTLAYSGKLVSPKWKNFKGLKLQWRD

Dog_MondoA MAADVFMCSPRRPRSRGRPMLLKPQVP-EDDDDSDTDEPSPPPACASAAPARAHASAAPPPPRAGPG----------------REEPP-----RRQQIIHSGHFMVSSPHREHPPKKGYDFDTVNKQTCQTYSFGKTSSCHLSIDASLTKLFECMTLAYSGKLVSPKWKNFKGLKLQWRD

Mouse_MondoA MAADVFMCSPRRPRSRGRSVLLKPQVP-EDDDDSDTDEPSPPPPSGVATSARAHASAAPLPPRAGPG----------------REEPP-----RRQQIIHSGHFMVSSPHREHPPKKGYDFDTVNKQTCQTYSFGKTSSCHLSIDASLTKLFECMTLAYSGKLVSPKWKNFKGLKLQWRD

Rat_MondoA MAADVFMCSPRRPRSRGRSVLLKPQVP-EDDDDSDTDEPSPPPPSGVATSARAHASAAPLPPRAGPG----------------REEPP-----RRQQIIHSGHFMVSSPHREHPPKKGYDFDTVNKQTCQTYSFGKTSSCHLSIDASLTKLFECMTLAYSGKLVSPKWKNFKGLKLQWRD

Guinea_pig_MondoA MAADVFMCSPRRPRSRVRSVLLKSPVP-EDDDDSDTDEPSPPPASGSASVARAHASAAPLPPRSGSG----------------REEPP-----RRQQIIHSGHFMVSSPHREHPPKKGYDFDTVNKQTCQTYSFGKTSSCHLSIDASLTKLFECMTLAYSGKLVSPKWKNFKGLKLQWRD

Opossum_MondoA MAADVFMCSPRRPGGGARPVLLKAPAPDDDDDDSDTDEPAVAATAAAAAPAGSGPSSGPARPRPGPAATAGGPDDDEDDEAEDEEGAPGERRRGRQQIIHSGHFMMSSPHREHPPKKGYDFDTVNKQTCQTYSFGKTSSCHLSIDASLTKLFECMTLAYSGKLVSPKWKNFKGLKLQWRD

^2

<<< >>> MCR4 <<< >>> MCR6 <<< >>> MCR5

LID β--> <<<< >>>> GRACE

Human_CHREBP KIRLNNAIWRAWYIQYVKRRKSPVCGFVTPLQGP-EADAHRKPEAVVLEGNYWKRRIEVVMREYHKWRIYYKKRLRKP--------SREDDLLAPKQAEGRWPPPEQWCKQLFSSVVPVLLGDPEEEPGGRQLLDLNCFLSDISDTLF-TMTQSGPSPLQLPPEDAYVGNADMIQPDLTP

Chimpanzee_ChREBP KIRLNNAIWRAWYIQYVERRKSPVCGFVTPLQGP-EADAHRKPEAVVLEGNYWKRRIEVVMREYHKWRIYYKKRLRKP--------SREEDLLAPKQAEGRWPPPEQWCKQLFSSVVPVLLGDPEEEPGGRQLLDLNCFLSDISDTLF-TMTQSGPSPLQLPPEDAYVGNADMIQPDLTP

Vervet-AGM_ChREBP KIRLNNAIWRAWYIQYVERRKSPVCGFVTPLQGP-EADAHRKPEAVVLEGNYWKRRIEVVMREYHKWRIYYKKRLRKS--------SREEDLLAPKQAEGGWPPPEQWCKQLFSSVVPVLLGDPEEEPGGRQLLDLNCFLSDISDTLF-TMTQSGPSPLQLPPEDAYVGNADMIQPDLTP

Pig_ChREBP KIRLNNAIWRAWYIQYVERRKSPVCGFVTPLQGP-EADEHRKPEAVILEGNYWKRRIEVVMREYHKWRIYYKKRLRKS--------SREGDLLAPKQAEGGWQPPERWCEQLFTSVVPVLLGGPEEEPGGRQLLDLDCFLSDISDTLF-TTTQPSPTPLQLPPEDAYVGNADMIQPDLTP

Dog_ChREBP KIRLNNAIWRAWYIQYVERRKSPVCGFVTPLQGP-EADEHRKPEAVVLEGNYWKRRIEVVMREYHKWRIYYKKRLRKS--------SREGDLLAPKQAEGDWQPPERWCEQLFSSVVPVLLGGPEKEPGGRQLLDLDCFLSDISDTLF-TMTQPSSAPLQLPPEDAYVGNADMIQPDLTP

Bushbaby_ChREBP KIRLNNAIWRAWYIQYVERRKSPVCGFVTPLQGP-EADAHRKPEAVVLEGNYWKRRIEVVMREYHKWRIYYKKRLRKS--------SREGDLLAPKQADGRWPPPERWCEQLFSSVVPVLLGGPEEEPGGRQLLDLECFLSDISDTLF-TMTQPSPSPLQLPPEDAYVNNADMIQPDLTP

Mouse_ChREBP KIRLNNAIWRAWYIQYVQRRKSPVCGFVTPLQGS-EADEHRKPEAVILEGNYWKRRIEVVMREYHKWRIYYKKRLRKS--------SREGDFLAPKQVEGGWPPPERWCEQLFSSVVPVLLGGSEEEPGGRQLLDLDCFLSDISDTLF-TMTQPSPSSLQLPPEDAYVGNADMIQPDLTP

Rat_ChREBP KIRLNNAIWRAWYIQYVQRRKSPVCGFVTPLQGS-EADEHRKPEAVVLEGNYWKRRIEVVMREYHKWRIYYKKRLRKS--------SREGDFLAPKQVEGGWPPPERWCEQLFSSVVPVLLGGSEEEPGGRQLLDLDCFLSDISDTLF-TMTQPSPSSLQLPSEDAYVGNADMIQPDLTP

Guinea_pig_ChREBP KIRLNNAIWRAWYIQYVQRRKSPVCGFVTPLQGP-EADEHRKPEAVVLEGNYWKRRIEVVMREYHKWRIYYKKRLRKS--------IREGDLLAPKQAEGGWPPPERWCQQLFSSVVPVLLGSPEEEPGGRQLLDIDCFLSDISDTLF-TMTQPNSSPLQLPPEDAYISNADMIQPDLTP

Opossum_ChREBP KIRLNNAIWRAWYIQYVQRRKSPVCGFVTPLQGS-EADEHRKPEAVVLEGNYWKRRIEVVMQEYHKWRIYYKKRLRKS--------SREGDFLAPKQAEGGWRPPERWCEQLFSSVVPVLLGGPEEEPGGRQLLDLDCFLSDISDTLF-TMTQPSPAPLQLPPDDAYIGNADMIQPDLTP

^1 ^0 ^1 ^0 ^1

Human_MONDOA KIRLNNAIWRAWYMQYLEKRKNPVCHFVTPLDGSVDVDEHRRPEAITTEGKYWKSRIEIVIREYHKWRTYFKKRLQQHKDEDLSSLVQDDDMLYWHKHGDGWKTP-----------VPM-----EEDP----LLDTDMLMSEFSDTLFSTLSSHQPVAWPNPREIAHLGNADMIQPGLIP

Chimpanzee_MondoA KIRLNNAIWRAWYMQYLEKRKNPVCHFVTPLDGSVDVDEHRRPEAITTEGKYWKSRIEIVIREYHKWRTYFKKRLQQHKDEDLSSLVQDDDMLYWHKHGDGWKTP-----------VPM-----EEDP----LLDTDMLMSEFSDTLFSTLSSHQPVAWPNPREIAHLGNADMIQPGLIP

Vervet-AGM_MondoA KIRLNNAIWRAWYMQYLEKRKNPVCHFVTPLDGSVDVDEHRRPEAITTEGKYWKSRIEIVIREYHKWRTYFKKRLQQHKDEDLSSLVQDDDMLYWHKHGDGWKTP-----------VPM-----EEDP----LLDTDMLMSEFSDTLFSTLSSHQPVAWPNPREIAHLGNADMIQPGLIP

Bushbaby_MondoA KIRLNNAIWRAWYMQYLEKRKNPVCHFVTPLDGSVDVDEHRRPEAITTEGKYWKSRIEIVIREYHKWRTYFKKRLQQHKDEDLSSLAQDDDMLYWHKHRDGWKTP-----------VPM-----EEDP----LLDTDMLMSEFSDTLFSTLSSHQPVAWPNPREIAHLGNADMIQPGLIP

Pig_MondoA KIRLNNAIWRAWYMQYLEKRKNPVCHFVTPLDGSVEVDEHRRPEAITTEGKYWKSRIEIVIREYHKWRTYFKKRLQQHKDEDLSSLAQDDDMLYWHKHGDGWKTP-----------VPM-----EEDT----LLDTDMLMSEFSDTLFSTLSSHPPVAWPNPREIAHLGNADMIQPGLMP

Dog_MondoA KIRLNNAIWRAWYMQYLEKRKNPVCHFVTPLDGSVEVDEHRRPEAITTEGKYWKSRIEIVIREYHKWRTYFKKRLQQHKDEDLSSLAQDDDMLYWHKRGDGWKTP-----------VPM-----EEDP----LLDTDMLMSEFSDTLFSTLSSHQPVAWPNPREIAHLGNADMIQPGLIP

Mouse_MondoA KIRLNNAIWRAWYMQYLEKRKNPVCHFVTPLDGSVDVDEHRRPEAITTEGKYWKSRIEIVIREYHKWRTYFKKRLQQHKDEDLSSLAQDDDMLYWHKHGDGWKTP-----------VPM-----EEDS----LLDTDMLMSEFSDTLFSTLSSHQPVAWPNPREIAHLGNADMIQPGLIP

Rat_MondoA KIRLNNAIWRAWYMQYLEKRKNPVCHFVTPLDGSVDVDEHRRPEAITTEGKYWKSRIEIVIREYHKWRTYFKKRLQQHKDEDLSSLAQDDDMLYWHKHGDGWKTP-----------VPM-----EEDS----LLDTDMLMSEFSDTLFSTLSSHQPVAWPNPREIAHLGNADMIQPGLIP

Guinea_pig_MondoA KIRLNNAIWRAWYMQYLEKRKNPVCHFVTPLDGSVEVDEHRRPEAITTEGKYWKSRIEIVIREYHKWRTYFKKRLQQHKDEDLSSLAQDDDMPYWHKHGDGWKTP-----------VPM-----EEDP----LLDTDMLMSEFSDTLFSTLSSHQPVAWPNPREIAHLGNADMIQPGLIP

Opossum_MondoA KIRLNNAIWRAWYMQYLEKRKNPVCHFVTPLDGSVEVDEHRRPEAITTEGKYWKHRIEIVIREYHKWRTYFKKRLQKHKDEDLSSLVRDDDLLFWHKSGDGWDTP-----------VPM-----EEDP----LLDTDMLMSEFSDTLFSTLSSHQPMAWPNPREIAHLGNADMIQPGLIP

^1 ^0 ^1 ^0 ^1

<<<

<<<< >>>> Proline-rich

Human_CHREBP LQPSLDDFMDI----SDFFTNSRL---PQPPMPSNFPEP-PSFSPVVDSLFSSGTLGPEVP-----PASSAMTHLSGHSRLQARNSCPGPLDSSAFLSSDFLLPEDPKPRLPP---PPVPPPLLHY---------PPPAKVPGLEPCPPPPFPPMAPP-TALLQEEPLFSPRFPFPTVPP

Chimpanzee_ChREBP LQPSLDDFMDI----SDFFTNSRP---PQPPMPSNFPEP-PSFSPMVDSLFSSGTLGPEVP-----PASSAMTHLSGHSRLQARNSCPGPLDSSAFLSSDFLLPEDPKPRLPT---PPVPPPLLHY---------PPPAKVPGLEPCPPPPFPPMAPP-TALLQEEPLFSPRFPFPTVPP

Vervet-AGM_ChREBP LQPSLDDFMDI----SDFFTNSRP---PQPPMPSNFPEP-PNFSPVVDSLFSSGTLGLEVP-----PASSAMTHLSGHSRLQARNSCPGPLDSSAFLSSDFLLPEDPKPRLPP---PSVPPPLLHY---------PPPAKVPGLEPCPPPPFPPMAPP-TALLQEEPLFSPRFPFPTVPP

Pig_ChREBP LQPSLDDFMEI----SDFFTNYRP---PQTPTPSNFSEP-PSFGPMADPVLSSGVLGSELP-----PASLGMTQLSGHNRLQARSSCPGPLDSSAFLSSDFLLPEDPKPKLPPQAQAPAAPPLIQY---------PSPTKGLGLEPCPPPPFPPMVPP-PAMLQEEPLFSPRFSFPTIPP

Dog_ChREBP LQPSLDDFMEI----SDFFTSYRP---PQTPTPSHFPEP-PSFGPMADPFFSSGILGSEVP-----PASSGVTHLSGHNRLQARSSCPGPLDSSAFLNSDFLLPEDPKPKLPP---PPVPPPLFQY---------PALAKGPGLEPCAPPTFPPMAPP-PALLQEEPLFSPRFPFSTIPP

Bushbaby_ChREBP LQPSLDDCMEI----SDFFTNYRP---PQTPTPSNFLEP-PSFSPMADSFFSSGILGPEVT-----AASSGLTHLSGHGRLQARNSSPGPLDSSTFLSSEFLLPEDPKPRLPP---PLTPPPLFQY---------SPPAKALGLEPCPPPPFPPMAPP-PAVLQEESLFSPRLPFPTVPP

Mouse_ChREBP LQPSLDDFMEI----SDFFTNYRP---PQTPTSSNYIES-PSFGPMADSLFSSGILAPEMPSPASSSSSSGMTPHSGNTRLQARNSCSGPLDPNPFLSSEFLLPEDPKTKIPP---APGPTPLLPF---------PTPVKVHGLEPCTPSPFPTMAPP-PSLLPEESLLSARFPFTSAPP

Rat_ChREBP LQPSLDDFMEI----SDFFTNYRP---PQTPTSSNFPEP-PSFGPMADSLFSGGILGPEMPSPASASSSSGMTPLSGNTRLQARNSCSGPLDPSTFPSSEFLLPEDPKTKMPP---APVPTPLLPY---------PGPVKVHGLEPCTPSPFPTMAPP-PALLSEEPLFSARFPFTTVPP

Guinea_pig_ChREBP LQPSLDDFMES----SDFFTNYRL---PQTPTASNFMEP-PSFTPMADSGFGSGILAPEAP-----PASSGLPHIPGQSRLQARNSCPGPLDPSAFRSSDFFLPEEPKAKLPP---PSGPPHLLQY---------PAPDKVPGLQPCTPSSFPPVASA-PALLQEEPRFSARFPFPAAPQ

Opossum_ChREBP LQPSLDDFMDI----SEFFINYRA---SSTLVS--FSEP-L?FSSMADPLFSGGFLAPESQ-----AAPPGVPLLLG-SRVPARSSCSAPLDSPAFLSPDFLLPEDPKPK-PP-------QALLHF---------PAPPKAPGLEP----------------------WARR----GVPE

^1 ^0

Human_MONDOA LQPNL-DFMDTFEPFQDLFSSSRSIFGSMLPASASAPVPDPNNPPAQESILPTTAL-PTVSLPDSLIAPPTAPSLAH----MDEQGC----EHTSRTEDPFIQPTDFGPSEPP---LSVPQPFLPVFTMPLLSPSPAPPPISPVLPLVPPPATALNPPAPPTFHQPQKFAGVNKAPSVIT

Chimpanzee_MondoA LQPNL-DFMDTFEPFQDLFSSSRSIFGSMLPASASAPVPDPNNPPAQESILPTTAL-PTVSLPDSLIAPPTAPSLAH----MDGQGC----EHTSRTEDPFIQPTDFGPSEPP---LSVPQPFLPVFTMPLLSPSPAPPPISPVLPLVPPPATALNPPAPPTFHQPQKVAGVNKAPSVIT

Vervet-AGM_MondoA LQPNL-DFMDTFEPFQDLFSSSRSIFGSMLPASASAPVPDPNNPPAQESVLQTTAL-PTVSLPDSLIAPPTAPPLAH----VDGQSC----EHTSRAEDPFIQPTDFGPSEPP---LSVPQPFLPVFTMPLLSPSPAPPPVSPVLPLVPPPATALNPPAPPTFHQPQKFAGVSKVPSVIT

Bushbaby_MondoA LQPNL-DFMDTFEPFQDLFSSSRSIFNSLLPAPASASAPDPNSTPAQESILPTTTL-PTGSLPDNLIVPPTAPSLDP----TDGQGC----ERVSRTGDPFIQPSDFGPSEPS---LSIPQSFLPVFTVPLLSPSPAPAPMSPALPLV-PPATALNPPAPSAFLQPQKSAGVSKSPSVIT

Pig_MondoA LQPNL-DFMDTFEPFQDLFSSSRSIFGSMLPAPASAAAPDPNSPPAQESILPTTAL-PTSSLPDSFIAPPVVTALDP----TNGQGC----EHTSQPGDPFVQPAELGPSAPP---LTVPQSFLPVFTMPLLSPSPAPAPVSPALHLGPPPATALSPPTPPAFLQPQKFAEVSKSPSVIT

Dog_MondoA LQPNL-DFMDTFEPFQDLFSSSRSIFGSMLPAPVSAPAPDPNSPPAQETILPTSAL-PTVSLPDSLIAPPAATALDP----TDRQGC----ERAPRPGDPFIQPTDFGPPAPP---LNVPQPFLPVFTMPLLSPSPAPAPTSPALPLAPPPATALSPSAPPTFLHP-KFAGASKSPSVIT

Mouse_MondoA LQPNL-DFMDTFEPFQDLFSSSRSIFGSMLPPPSSLPAADPSSPPSQGNILPNTAL-PPASLPNSLITSSAAPSLDP----TEGQGC----ERTSQTVDPFIQPADFGPSEPP---LSVPQPFLPVFTMTLLSPGPAPAPVPTALPLVPSPAPTLNPPTPPAFLQPQKFAGVSKSTPVIT

Rat_MondoA LQPNL-DFMDTFEPFQELFSSSRSIFGSMLPPPTSVPAPDPSSPPSQGNILPNPAL-PPVSLPNSLIASSAAPSLDP----TEGQGC----ERTSQSVDPFIQPADFGPSEPP---LSVPQPFLPVFTMTLLSPGPAPAPVPTALPLVSPPAPTLNPPTPPAFLQTQTFAGVSTSSPVIT

Guinea_pig_MondoA LQPNL-DFMDTFEPFQDLFSASRAIFGSVPPVPASAPVPDASSSPAEDSILPATAL-PTVSLPDSLIAPSVGPPLQP----SASQGC----DRASQPGDPFLQSPDFTPSESP---LGVAQPFLPVFPMPLLSPGPTQTPSPPGIPSVPPPGPALTPPAPPAFQQLPRFSGLSKPPPVIT

Opossum_MondoA LQPNF-DFMDTFEPFQDLFSSSRSIFGPTLAGPTVSTATDPSSPPAQGPILSAGAL-PTVSLPDSLIVTPGPASLSP----TERRSC----ERGPRLGGPFIQPTDFGSPEPQ---LSGPQTFLSVFPGSLLSPSPAPTPSSPAMPLVPSPGATLSAQAPSTFLQSQKFE-VGKSSSVIT

^1 ^0

Proline-rich

Human_CHREBP APGVSPL---PAPAAFPPTP-QSVPS--PAPTPFPIELLPLGYSEPAFG------PCFSM-----PRGKPPAPSPRGQKAS-PPTLAPATASPP--TTAGSNNPCLTQLLTAAKPEQALEPP-------------------LVSSTLLRSPGSPQETVPEFP--CTFLPPTPAPTPPRPP

Chimpanzee_ChREBP APGVSPL---PAPAAFPPTP-QSVPS--PAPTPFPIELLPLGYSEPTFG------PCFSM-----PRGKPPAPSPRGQKAS-PPTLAPATASPP--TTAGSNNPCLTQLLTAAKPEQALEPP-------------------LVSSTLLQSPGSPQETVPEFP--CTFLPPTPAPTPPRPP

Vervet-AGM_ChREBP APGVSPL---PAPAAFPPTP-QSVPS--PAPTPFPIELLPSGYSEPAFG------PCFSV-----PRGKPPAPSPRGQKAS-PPTLAPATASPP--TTVGSNNPCLTQLLTAAKPEQALEPP-------------------LVSSTLLRSPGSPQETVPEFS--CTFLPPTPAPTPPRPP

Pig_ChREBP ALGVSPL---STPTAFPATA-QPGPG--PTPAPFPIDLQRLGYSEPPFG------PHFMVPQSARPRGKPPTLSPRGRKPS-APTLAPATASPT--ATAGGNNPCLTQLLTAAKPEQALEPP-------------------LASGPLLRSPGSPQETVPDFP--CTFFPPTLASTPPRPP

Dog_ChREBP APGVSPL---PAPTAFPPTP-QPGPG--PAPPPFPIDLLPSGYLEPQFG------PHFTVPQSMRPRGRPPTPSPRGRKPS-PPTLAPATANST--ATAGGSNPCLTQLLTAAKPEQTLEPP-------------------LVSSTLLRPSGSPQEMAPEFP--CTFLPPTPAPTPPRPP

Bushbaby_ChREBP GLGVSPL---PVPTPFPPTP-QPTPG--PTTSPFPINLMATGCPEPLFG------PCFSTSQGTWPRGKPPS---RGRKAR-PPTLAPATASPTVAATAGGSNPCLTQLLTAAKPEQALEPP-------------------LVSSALLRPPGSPQETVPEFP--CTFFPPTPAPTPARPP

Mouse_ChREBP APGVSTL---PAPTTFVPTP-Q--PG--PGPVPFSVDHLPHGYLEPVFG------PHFTVPQGMQPRCKPSSPSPGGQKAS-PPTLASATASPTATATARDNNPCLTQLLRAAKPEQALEPP-------------------TMPGTLLRPPESPQDTVSEIPRARAFFPPIPAPTPPRPP

Rat_ChREBP APGVSTL---PAPTTFVPTP-QPGPG--PGPVPFPVDHLPHGYLEPVFG------PHFTVPQGVQPRCKPCSPPPGGRKAS-PPTLTSATASPTATATARDNNPCLTQLLRAAKPEQVLEPS-------------------TVPSTLLRPPESPQDAVPEIPRVRAFYPPIPAPTPPRPP

Guinea_pig_ChREBP ARGASPL---PAPTAFPATPVQPIPGPVPGPTPFPIDPLSSGYPEPSFS------SPFAVPQGVQPRGKPLGPPSRERKASTTPTLAS--------ATTQGSNPCLTQLLRAAKTEQAVEPP-------------------LVPSTLLRPPGSPQETVPEFP--HVFFPLTLASIPPRPP

Opossum_ChREBP GLGGSGL---GA---------------------------SGGGESGLFSWVCSWFFFFAA-----PRGRRPSPA----------------------ASAGGSNPCLTQLLTAPKPEHTLEAP-------------------PSSAT-LGPSGSPKPGPRDFP--GAFFSP-----PAQPA

^1 ^0

Human_MONDOA HTASATLTHDAPATTFSQSQGLVITTHHPAPSAAPCGLALSPVTR--------------PPQPRLTFVHPKPVSLTGGRPKQPHKIVPAPK-PEP-VSLVLKNARIAPAAFSGQPQAVIMTSGPLKREGMLASTVSQSNVVIAPAAIARAPG-----VPEFHSSILV-TDLGHGTSSPPA

Chimpanzee_MondoA HTASATLTHDAPATTFSQSQGLVITTHHPAPSAAPCGLALSPVTQ--------------PPQPRLTFVHPKPVSLTGGRPKQPHKIVPAPK-PEP-VSLVLKNARIAPAAFSGQPQAVIMTSGPLKREGMLASTVSQSNVVIAPAAIARAPG-----VPEFHSSILV-TDLGHGTSSPPA

Vervet-AGM_MondoA HTASATLTHDASATTFSQSQGLVITTHHPAPSAAPCGLALSPIPR--------------PPQPRLTFVHPKPVSLTGGRPKQPHKIVPAPK-PEP-VSLVLKNTCITPAAFSGQPQAVIMTSGPLKREGILASTVSQSNVVIAPAAIARAPG-----VPEFHSSILV-TDLSHGTNSPPA

Bushbaby_MondoA HTASATLTHDASA-TFSQSQGLLITTHHPAPSTAPCSLALSPITQ--------------SPAPRLTFVPPKPVSMTGGRPKQPPKIVPAPK-PEP-VSLVLKNACLAPAAFSGQPQAVIMTSGHLKREGMLASTVSQSNVVIAPAAIARAPG-----VTEFHSSILV-TDLSHSTSSQPG

Pig_MondoA HTASATLTHDASATTFSQSQGLIITTRYPTPSMSACGLALSPVTRP---------MTVGPPQPRLTFVHPKPVSLPGVRHKQPPKIVPAPK-PEP-VSLVLKNARIAPAAFSGPPQAVIMTSGPLKREGMLASTVSQSNMVITPAAITRAPG-----VTEFHSSILV-TDLGHSTGSQPA

Dog_MondoA HTASATLTHDASATTFSQSQGLVITTHHPTPSVSPCGLALPPATRP---------PTAGPPQPCLTFVHPKPVSLTGGRPKQPPKIVPAPK-PES-VSVVLKNACIAPAAFSGQPQAVIMTSGPLKREGMLASTVSQSNMVLTPTAIARGPG-----VTEFHSGILV-TDLGHTTSSQPA

Mouse_MondoA HTASATLTHDASATTFSQNQGLVITAHHPTPSSSPCALALSPVPQP---------PAVGPPQPHLTFIHPKPVSLTGVRHKQPPKIVPAPK-PEP-VSLVLKNACIAPAAFSGQPQKVIMTSAPLKREGILASTVSPSNVVIASAAITRASG-----VTEF---------LSHSTSSQPS

Rat_MondoA HTASATLTHDASATTFSQSQGLVITAHHPTPSPSPCGLALSPIPQP---------PAVGPPQPHLTFIHPKPVSLTGVRHKQPPKIVPAPK-PEA-VSLVLKSACIAPAAFTGQPQKVIMTSAPLKREGILASTVSPSSVVIASAAITRASG-----VTEF---------LSHSTSSQPS

Guinea_pig_MondoA HTASTTLTHAAATTTFSQSQGLVIAAPHPPPSPSPCPLTLSPVPQP---------PAIGSSQTRLTFVHPKPVSLTGGRPKQPLKIVPAPK-PES-MSLVLKSTHLTPAAFPGHPQAVIMTSGPLKREGMLASTVSQPGVVITPASIPRAPG-----VTEFHSGILV-TDLGHSSSSQPA

Opossum_MondoA HTASATLTHDASATTFSQSQGLVLTAQQPAPPGPPCSLTV-----P---------PSVGPTQSRLTFVHPKSVPLASGRAKQPPKIVPAPKPPEP-VSLVLKNAYITPAAFSGQPQAVIVTPGLLKREGVLTSTISQPNVVIA-----RAPG-----VTEFHSGILVAADLGHATSSQPS

^1 ^0

<<<< >>>> bHLH-Zip <<<<

Human_CHREBP P-----GPATLAPSRPLLVPKAERL-----SPPAPS-GSERRLSGDLSSMPGPGTLSVRVSPPQPILSR-GRP-----DSNKTENRRITHISAEQKRRFNIK-LGFDTLHGLVSTLSAQPSLKVSKATTLQKTAEYILMLQQERAGLQEEAQQLRDEIEELNAAINLCQQQLPATGVPIT

Chimpanzee_ChREBP P-----GPATLAPSRPLLVPKAERL-----SPPAPS-GSERRLSGDLSSMPGPGTLSVRVSPPQPILSR-GRP-----DSNKTENRRITHISAEQKRRFNIK-LGFDTLHGLVSTLSAQPSLKVSKATTLQKTAEYILMLQQERAGLQEEAQQLRDEIEELNAAINLCQQQLPATGVPIT

Vervet-AGM_ChREBP P-----GPATLAPSRPLLVPKAEGL-----SPPALS-GSERRLSGDLSSMPGPGTLSVRVSPPQPILSR-GRP-----DSNKTENRRITHISAEQKRRFNIK-LGFDTLHGLVSTLSAQPSLKVSKATTLQKTAEYILMLQQERAGLQEEAQQLRDEIEELNAAINLCQQQLPATGVPIT

Pig_ChREBP P-----GPATLAPPRPLIVPKVERL-----SPPAPS-GGERRLSGELNSAPGPGTLSIHISPSQPIPGRGGRP-----D--KTENRRITHISAEQKRRFNIK-LGFDTLHGLVSTLSTQPNLKMSKATTLQKTAEYITMLQQERAAKQEEAQQLRDQIEELNAAINLCQQQLPATGVPIT

Dog_ChREBP P-----GPATLAPPRPLIVPKAERL-----SPPAHS-GGKRRLSGELSSMQGLGTLSVHVSPPQPILSR-GRP-----DNNKTENRRITHISAEQKRRFNIK-LGFDTLHGLVSTLSAQPSLKVSKATTLQKTAEYIAMLQQERAAMQEEAQQLRDQIEELNAAINLCQQQLPATGVPIT

Bushbaby_ChREBP P-----GPATLAPSRPLIVPKAERL-----SPPTPS-GSERRLSGELNSTPSPGTLSIRISPPQPIIHR-GRA-----DNNKTENRRITHISAEQKRRFNIK-LGFDTLHGLVSTLSAQPSLKVSKATTLQKTAEYIVMLQQERAAMQEEAQQLRDEIEELNAAINLCQQQLPATGVPIT

Mouse_ChREBP P-----GPATLAPPRSLVVPKAERL-----SPPASS-GSERRLSGDLNSIQPSGALSVHLSPPQTVLSR-GRV-----DNNKMENRRITHISAEQKRRFNIK-LGFDTLHGLVSTLSAQPSLKVSKATTLQKTAEYILMLQQERAAMQEEAQQLRDEIEELNAAINLCQQQLPATGVPIT

Rat_ChREBP P-----GPATLAPPRSLVVPKAERL-----SPPASS-GSERRLSGDLNSIQPPGALSVHLSPPQTVLSR-GRV-----DNNKMENRRITHISAEQKRRFNIK-LGFDTLHGLVSTLSAQPSLKVSKATTLQKTAEYILMLQQERAAMQEEAQQLRDEIEELNAAINLCQQQLPATGVPIT

Guinea_pig_ChREBP L-----GPATLAPPRPLIVPKAERL-----SPPAPS-GSERRLSEELNCVPALGALSVGVSPPPLVPCR-GRP-----DNSKTESRRITHISAEQKRRFNIK-LGFDILHGLVSTLSAQPNLKVSKATTLQKTAEYIIMLQQERAALQQEAQQLRDEIEELNAAINLCQQQLPATGVPIT

Opossum_ChREBP L-----TPATL-------VPKAERL-----SPPA-A-GHERRLSGELPPLPGPMGLGSRISPPQPTLGR-GRP-----D--KTENRRITHISAEERRRFQHQAWAFDTLHGLVSTLSAQPSLKVSKATTLQKTAEYIAMLQQERAAMQEEAQHLRDQIEELNAAINLCQQQLPATGVPIT

^1 ^0 ^0 ^2

Human_MONDOA PVSRLF-PSTAQDP----LGKGEQVPLHGGSPQVTVTGPSRDCPNSGQASPCASEQSPSPQSPQNNCS--GKS--DPKNVAALKNRQMKHISAEQKRRFNIK-MCFDMLNSLISNNSKL----TSHAITLQKTVEYITKLQQERGQMQEEARRLREEIEELNATIISCQQLLPATGVPVT

Chimpanzee_MondoA PVSRLF-PSTAQDP----LGKGEQVPLHGGSPQVTVTGPSRDCPNSGQASPCASEQSPSPQSPQNNCS--GKS--DPKNVAALKNRQMKHISAEQKRRFNIK-MCFDMLNSLISNNSKL----TSHAITLQKTVEYITKLQQERGQMQEEARRLREEIEELNATIISCQQLLPATGVPVT

Vervet-AGM_MondoA PISRLF-PSTAQDP----LGKGEQVQLHGGSPQVTVTGPSRDCPNSGQASPCASEQSPSPQSPQNNCS--GKS--DPKNVAALKNRQMKHISAEQKRRFNIK-MCFDMLNSLISNNSKL----TSHAITLQKTVEYITKLQQERSQMQEEARRLREEIEELNATIISCQQLLPATGVPVT

Bushbaby_MondoA PVSRLFSPGTIQDS----LVKGEQVSLPGGSPQVPVTGPSRDGSNSGQASPCALEQSPSPQSPQNNCS--GKS-ADPKNMAALKNRQMKHISAEQKRRFNIK-MGFDTLNSLISNSSKL----TSHAITLQKTVEYITKLQQERSQMQEEARRLRDEIEELNATIISCQQLLPATGVPVT

Pig_MondoA PVSRLFSPSTVPDS----LVKGEQAPLHGGAPSVPATGPSRDCPNSGQASPCASEQSPSPQSPQNNCS--GKSTADLKNVAALKNRQMKHISAEQKRRFNIK-MGFDTLNSLISNNTKL----TSHAITLQKTVEYITKLQQERSQMQEEARRLREEIEELNATILSCQQLLPATGVPVT

Dog_MondoA PVSRLFSPSTVQDS----LVKGEQ---------VPATGSSRDCPNSGQASPCASEQSPSPQSPQNNCS--GKS-ADPKNVAALKNRQMKHISAEQKRRFNIK-MGFDTLNSLISNNSKL----TSHAITLQKTVEYITKLQQERSQMQEEARRLREEIEELNATIISCQQLLPATGVPVT

Mouse_MondoA PVSRLFSPSTVQDS----LVKGEQVSLHGGSPQVPATGSSRDCPNSGQASPCPSEQSPSPQSPQNNCS--GKS-TDPKNVAALKNRQ-KHISAEQKRRFNIR-MGFNTLNSLISNNSKQ----TSHAITLQKTMEYITKLQQERMQMQEEARRLREEIEELNTTIISCQQLLPATGVPVN

Rat_MondoA PASRLFSPSAVQDA----LVKGEQAALHGG-PQAPATGSSRDCPNSGQASPCPSEQSPSPQSPQNNCS--GKS-TDPKNVAALKNRQ-KHISAEQKRRFNIR-MGFNTLNSLISNNSKQ----TSHAITLQKTMEYITKLQQERMQMQEEARRLREEIEELNTTIISCQQLLPATGVPVN

Guinea_pig_MondoA PVSRLFSPGIVQDS----LVKVEQAPLRGGSPQVPATGPGRDGLNSGQASPCASEQSPSPQSPQNNCS--GKS-ANPKNMSVLKNQQLKHISAEQKRRFNIK-MGFNTLNSLISNNSKP----SSHAITLQKTVEYITKLQQERSQMQEEARRLREEIEELNATIISCQQLLPASGVSVT

Opossum_MondoA PASRLFSPSAVQDS----LVKGEQISLHGSSSQMASPSSSRDCPNSGQASPCASEQSPSPQSPQ-NCS--GKSATDPPNVAAFKNRRMKHISAEQKRRFNIK-IGFGTLNSLISNNCKL----TSHAITLQKTVEYIAKLQQERSQMQDEAQRLREEIEELNTTIISCQQQLPATGVPVT

^1 ^0 ^0 ^2

>>>> DCD <<<<

Human_CHREBP HQRFDQMRDMFDDYVRTRTLHNWKFWVFSILIRPLFESFNGMVSTASVHTLRQTSLAWLDQYCSLPALRPTVLNSLRQLGTSTSILTDPGRIPEQATRAVTE-----GTLGKPL

Chimpanzee_ChREBP HQRFDQMRDMFDDYVRTRTLHNWKFWVFSILIRPLFESFNGMVSTASVHTLRQTSLAWLDQYCSLPALRPTVLNSLRQLGTSTSILTDPGRIPEQATRAVTE-----GTLGKPL

Vervet-AGM_ChREBP HQRFDQMRDMFDDYVRTRTLHNWKFWVFSILIRPLFESFKGMVSTASVHTLRQTSLAWLDQYCSLPALRPTVLNSLRQLGTSTSILTDPGRIPEQATRAVTE-----GTLGKPL

Pig_ChREBP HQRFNQMRDMFDDYVRTRTLHNWKFWVFSILIRPLFESFNGMVSTASLQSLRQTSLAWLDQYCSLPALRPTVLNSLRQLSTSTSILTDPDCIPEQATRAVTE-----GTLGKPL

Dog_ChREBP HQRFDQMRDMFDDYVRTRTLHNWKFWVFSILIRPLFESFNGMVSTASLQSLRQTSLAWLDQYCSLPALRPTVLNSLRQLSTSTSILTDPACIPEQATRAVTE-----GTLGKPL

Bushbaby_ChREBP HQRFDQMRDMFDDYVRTRTLHNWKFWVFSVLIRPLFESFNGMVSTASLHSLRQTSLAWLDQYCSLPALRPTVLNSLRQLSTSTSILTDPGCIPEQATRAVTE-----GTLGKPL

Mouse_ChREBP HQRFDQMRDMFDDYVRTRTLHNWKFWVFSILIRPLFESFNGMVSTASLHSLRQTSLAWLEQYCSLPALRPTVLNSLRQLSTSTSILTDPSLVPEQATRAVTE-----GTLGRPL

Rat_ChREBP HQRFDQMRDMFDDYVRTRTLHNWKFWVFSILIRPLFESFNGMVSTASLHSLRQTSLAWLDQYCSLPALRPTVLNSLRQLSTSTSILTDPSLVPEQATRAVTE-----GPLGRPL

Guinea_pig_ChREBP HQRFDQMRDMFDDYVRTRTLHNWKFWVFSILIRPLFESFNGMVSTASLHSLRQTSLAWLDQYCSLPALRPTVLNSLRQLSASTSILTDPSRVPEQATRAVTE-----GILGRPL

Opossum_ChREBP HQRFDQMRDMFEDYVRTRTLHNWKFWVFSLLIRPLFESFNGMVSTASLHSLRQTSLAWLDQYCSLPALRPTILSSLRQLSTSTCILTDPARVPEQATRAVTE-----GKLQHPS

^0 ^1

Human_MONDOA RRQFDHMKDMFDEYVKTRTLQNWKFWIFSIIIKPLFESFKGMVSTSSLEELHRTALSWLDQHCSLPILRPMVLSTLRQLSTSTSILTDPAQLPEQASKAVTRIGKRLGES----

Chimpanzee_MondoA RRQFDHMKDMFDEYVKTRTLQNWKFWIFSIIIKPLFESFKGMVSTSSLEELHRTALSWLDQHCSLPILRPMVLSTLRQLSTSTSILTDPAQLPEQASKAVTRIGKRLGES----

Vervet-AGM_MondoA RRQFDHMRDMFDEYVKSRTLQNWKFWIFSIIIKPLFESFKGMVSTSSLEELHRTALSWLDQHCSLPILRPMVLNTLRQLSTSTSILTDPAQLPEQASKAVTRIGKRLGES----

Bushbaby_MondoA RRQFDHMRDMFDEYVKSRTLQNWKFWIFSIIIKPLFESFRGMVSSSSLEELHQTALSWLEQHCSLPVLRPMVLNTLRQLSTTTSILTDPSQLPEQASEAVTRIGKRSGES----

Pig_MondoA RRQFDHMRDMFDEYVKSRTLQNWKFWIFSVIIKPLFESFKGVVSTSSLEELHRTALSWLDEHCSLPVLRPTVLNTLRHLSTTTSILTNPSQLPEQAAEAVTRIGKRPGES----

Dog_MondoA RHQFDHMRDMFDEYVKSRTLQNWKFWIFSIIIKPLFESFKGMVSTSSLGELHRTALSWLDQHCSLPVLRPTVLNTLRHLSTTTSVLTDPSQLPEQAAEAVTRIGKRSGES----

Mouse_MondoA CRQLDHMRDMFDEYVKSRTLQNWKFWIFSMIIKPLFESFKGMVSTSSLEEFHRTALSWLDQHCSLPVLRPMVLSTLRQLSTTTSILTDPSQLPEQASEAVTRMGKRSGES----

Rat_MondoA CRQFDHMKDMFDEYVKSRILQNWKFWIFSMIIKPLFESFKGMVSTSSLEEFHRTALSWLDQHCSLPVLRPMVLSTLRQLSTTTSILTDPSQLPEQASEAVTRIGKRSGES----

Guinea_pig_MondoA PRQVDHMTDMFDEYVKSRTLQNWKFWIFSIIIKPLFESFKGVVSTSSLEELHRTALSWLDQHCSLPVLRPMVLSTLRQLSTTTSILTDPSQLPEQASEAVTRVGPRSGES----

Opossum_MondoA RRQYDHMRDMFDEYVKSRTLQNWKFWIFSIIIKPLFESFKGMVSTTNLHELHQSALSWLDQHCSLPVLRPMVLLTLRQLSTTTAILTDPSRLPEQASEAVTRISKRTGDSQHP-

^0 ^1

**S10 Fig. Alignment of MondoA and ChREBP sequences from species that contain both complete coding sequences**. MondoA and ChREBP sequences were aligned by MAFFT at the codon level. A fasta fomated verion of this alignment is presented in S11 Fig. The alignment was translated to generate a protein alignment. Locations of introns are indicated by ^ with the number referring to the phase of the codon interrupted by the intron are indicated below the MondoA and MondoA sequences, with identical intron positions found for all orthologs. The initiation codon for the beta isoform of ChREBP (ChREBP- β) is indicated by β. Domains in the MondoA and ChREBP sequences: LID, low glucose inhibitory domain; GRACE, glucose responsive activation conserved element; Pro-rich, proline rich; bHLH/ZIP, beta helix loop helix ZIP domain; ZIP-like, ZIP-like domain Locations of the domains in the protein sequences are indicated above the sequences, with the boundaries indicated by >>>> and <<<<. Conserved Mondo conserved regions (MCR1-6) in the LID and Grace domains are shown above the sequences, with boundaries indicated by >>> and <<<.
